# Cyclic Ion Mobility of Isomeric New Psychoactive Substances Employing Characteristic Arrival Time Distribution Profiles and Adduct Separation

**DOI:** 10.1021/jasms.4c00127

**Published:** 2024-07-01

**Authors:** Marianna Nytka, Jiahao Wan, František Tureček, Karel Lemr

**Affiliations:** † Department of Analytical Chemistry, Faculty of Science, Palacký University, 17. Listopadu 12, 77146 Olomouc, Czech Republic; ‡ Department of Chemistry, University of Washington, Seattle, Washington 98195-1700, United States

## Abstract

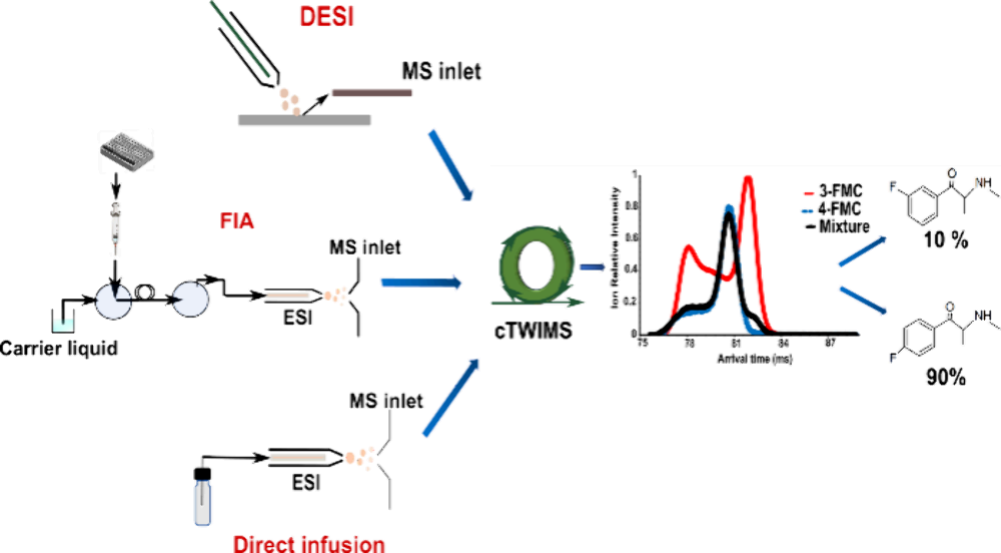

Analysis of new psychoactive substances (NPS), which is essential for toxicological and forensic reasons, can be made complicated by the presence of isomers. Ion mobility has been used as a standalone technique or coupled to mass spectrometry to detect and identify NPS. However, isomer separation has so far chiefly relied on chromatography. Here we report on the determination of isomeric ratios using cyclic ion mobility-mass spectrometry without any chromatographic separation. Isomers were distinguished by mobility separation of lithium adducts. Alternatively, we used arrival time distribution (ATD) profiles that were characteristic of individual isomers and were acquired for protonated molecules or fragment ions. Both approaches provided comparable results. Calculations were used to determine the structures and collision cross sections of both protonated and lithiated isomers that accurately characterized their ion mobility properties. The applicability of ATD profiles to isomer differentiation was demonstrated using direct infusion and flow injection analysis with electrospray of solutions, as well as desorption electrospray of solid samples. Data processing was performed by applying multiple linear regression to the ATD profiles. Using the proposed ATD profile-based approach, the relationships between the determined and given content of isomers showed good linearity with coefficients of determination typically greater than 0.99. Flow injection analysis using an autosampler allowed us to rapidly determine isomeric ratios in a sample containing two isomeric pairs with a minor isomer of 10% (determined 9.3% of 3-MMC and 11.0% of 3-FMC in a mixture with buphedrone and 4-FMC). The proposed approach is not only useful for NPS, but also may be applicable to small isomeric molecules analyzed by ion mobility when complete separation of isomers is not achieved.

## Introduction

New psychoactive substances (NPS) are a growing group of abused drugs. According to 2022 data, more than 1180 NPS have been identified in the last 15 years.^1^ Various analytical methods have been employed in NPS analysis, e.g., color and microcrystalline tests, infrared and Raman spectroscopy, nuclear magnetic resonance, mass spectrometry (MS), capillary electrophoresis, chromatography, and others have served to find synthetic cathinones in seized materials.^2^ NPS in biological fluids (blood, urine, oral fluid, etc.)^3,4^ and in hair^5,6^ have been analyzed by gas chromatography (GC-MS) and liquid chromatography (LC-MS) with various sample pretreatments. Both techniques are essential for the toxicological screening and confirmation of abused drugs.^7^ LC-MS has allowed the monitoring of NPS in pooled urine and urban wastewater (wastewater-based epidemiological studies).^8,9^ The large number of NPS, including many isomers, makes nontarget detection challenging. High resolution mass spectrometry is an important tool for solving this analytical task.^9,10^ Mass spectrometry has contributed to the identification of NPS in consumption products, the study of their metabolism and pharmacokinetics, and the analysis of authentic human samples.^9,11^ In addition to the commonly used MS in NPS’ chromatography, gas chromatography-infrared detection^12^ and gas chromatography-VUV spectroscopy^13^ have been successfully tested. Ambient ionization mass spectrometry has attracted attention, allowing the direct analysis of NPS.^14^ Supercritical fluid chromatography (SFC) is a promising alternative to gas and liquid chromatography. A fast chromatographic run (1.6 min) was sufficient to analyze a mixture of 15 NPS and separate isomers.^15^ For the detection and quantification of NPS in urine samples, the SFC-MS method was competitive with LC-MS.^16^

As a standalone technique, ion mobility spectrometry (IMS) has been employed to monitor NPS using reduced mobility values.^17−20^ Reduced mobility values were also combined with ambient and direct infusion mass spectrometry data to improve NPS confirmation and identification. Rapid screening of 35 NPS was performed by IMS with a ^63^Ni ion source and DART-QTOF (direct analysis in real time-quadrupole-time-of-flight).^21^ Similarly, the screening by ^63^Ni-IMS was followed by ESI-QTOF to confirm NPS in 24 seized samples.^22^ Drift tube ion mobility spectrometry expanded the detection tools for GC. Retention and drift time values were used for identification. Nine NPS representatives were quantified in oral fluid.^23^

The coupling of ion mobility-mass spectrometry (IM-MS) is a powerful analytical tool, but its application to the analysis of NPS has been limited so far. Compared to ^63^Ni-IMS, ESI-IM-MS with a drift tube provided better detection of some cathinones due to their improved ionization. Mass spectrometric data supported identification that was not limited only to the use of drift times. However, the mobility separation of isomers has been a problem for both compared techniques. For example, ESI-IM-MS gave one mobility peak for a mixture of mephedrone (4-MMC), buphedrone, and ethcathinone that remained unresolved.^24^ ESI-drift tube IM-MS was used to detect and identify four cathinones and five tryptamines in less than 1 min.^25^ Atmospheric pressure chemical ionization drift tube IM-MS achieved lower detection limits than ESI for three cathinones and three synthetic cannabinoids.^26^ However, isomeric compounds were not included in these two studies. LC-MS and LC-IM-MS were used to identify the metabolites of two synthetic cannabinoids after their incubation with rat and pooled human hepatocytes. The isomeric metabolites were chromatographically separated and showed different CCS values, but ion mobility separation of isomers was not reported.^27^ An LC-IM-MS method has been implemented in clinical routine for the analysis of 71 abused drugs, including NPS, in oral fluids. CCS values supported the identification, but IMS also increased the fragmentation of labile analytes.^28^ Ion mobility was integrated into an LC-IM-MS method applicable to the detection and quantification of fentanyl analogs in human urine. Ion mobility separation of metal cation adducts combined with data postprocessing by high-resolution demultiplexing was essential for isomer separation. Cations tested included, e.g., alkali and transition metals. No single cation was optimal for all groups of isomers. The use of several metal cations collectively was suggested to separate various isomers sufficiently.^29^ Rapid identification of isomeric cathinones differing in ring substitution was performed using ESI-trapped ion mobility-mass spectrometry. The mobilograms of all cathinones revealed the presence of two protomers (O- and N-protonated) whose mobility values allowed the detection of isomers. However, isomeric mixtures were not analyzed.^30^ The authors of neither this work nor the above cited works have used arrival time distribution (ATD) profiles that might be characteristic of isomers, as we demonstrated earlier for isomeric hyaluronan-derived oligosaccharides.^31,32^

**Figure 1:**
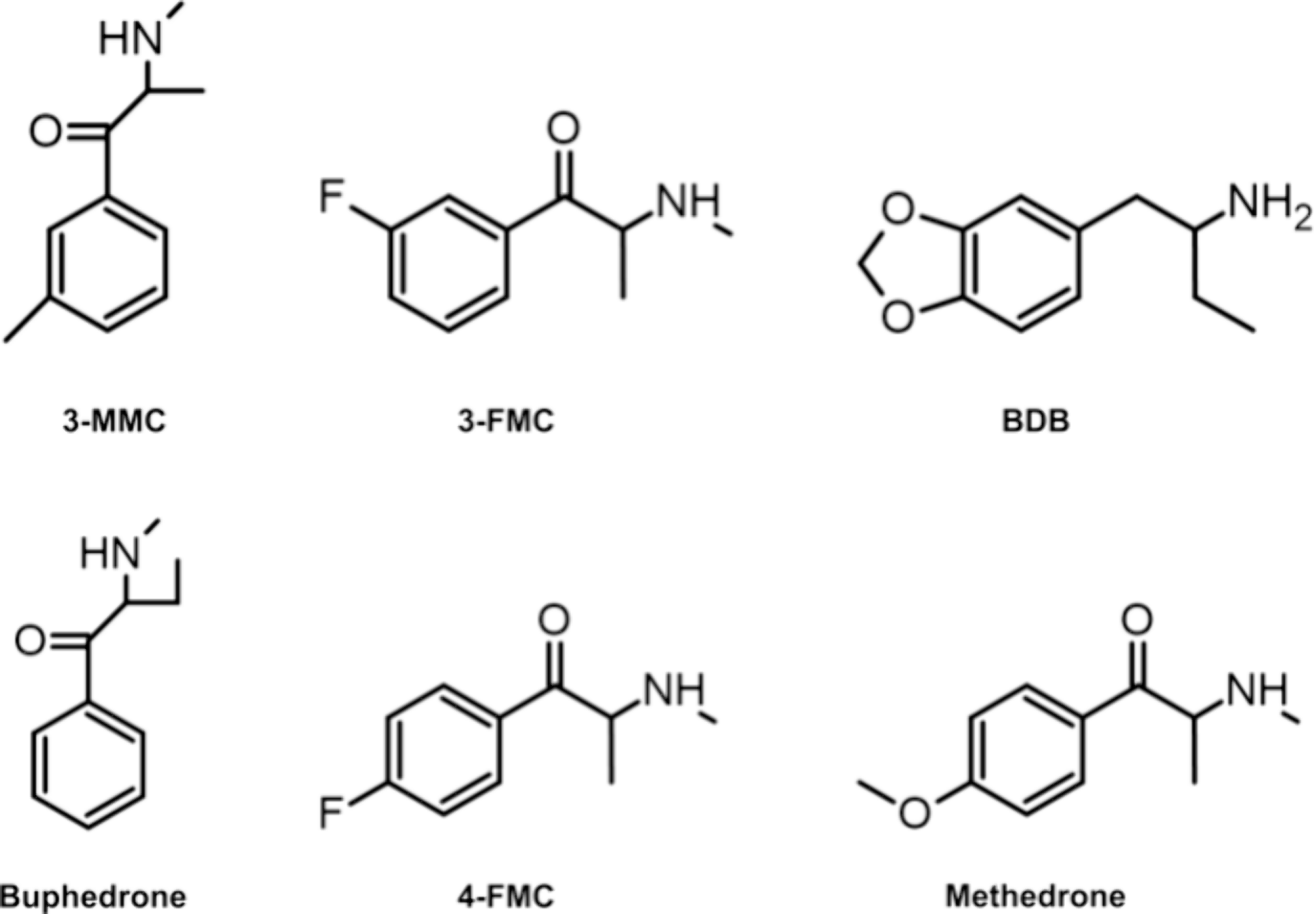
Investigated isomeric pairs: 3-methylmethcathinone (3-MMC) – buphedrone; 3-fluoromethcathinone (3-FMC) – 4-fluoromethcathinone (flephedrone, 4-FMC); 1,3-benzodioxolylbutanamine (BDB) – methedrone.

In the present work, we aimed to investigate cyclic traveling wave ion mobility (cyclic TWIM) of NPS isomers using three isomeric pairs (3-methylmethcathinone and buphedrone; 3-fluoromethcathinone and 4-fluoromethcathinone; 1,3-benzodioxolylbutanamine and methedrone, Figure 1). We evaluated the determination of isomeric ratios in mixtures employing characteristic ATD profiles in comparison with ion mobility separation of sodiated and lithiated molecules. For the analysis of isomeric mixtures, both the use of ATD profiles and the separation of Li^+^ adducts are suitable approaches. We demonstrated that ATD profiles are characteristic of small molecule isomers and confirmed this observation for both electrospray and desorption electrospray. We simplified the data processing by applying multiple linear regression instead of the previously used fitting.^32^ Our results may encourage the wider use of IM-MS in NPS analysis and ATD profiles in the ion mobility of isomers.

## Experimental Section

### Materials and Sample Preparation

Standards of hydrochlorides of 3-methylmethcathinone (3-MMC), 3-fluoromethcathinone (3-FMC), and buphedrone, 4-fluoromethcathinone (4-FMC), 1,3-benzodioxolylbutanamine (BDB), methedrone (see Figure 1 for structures) were purchased from Cayman Pharma (Neratovice, Czech Republic) and Lipomed AG (Arlesheim, Switzerland), respectively. LC-MS grade methanol and water were bought from Fisher Chemical (Fisher Scientific, U.K.). Sodium nitrate (p.a.) was provided by Penta (Ing. P. Švec, Czech Republic) and propan-2-ol (LC-MS grade), lithium chloride (≥99.0%, BioXtra), sodium hydroxide (p.a.), formic acid (≥98%), *N*-ethylaniline, acetaminophen, caffeine, sulfaguanidine, and alprenolol by Sigma-Aldrich (Prague, Czech Republic).

Stock and working solutions of individual analytes were prepared at the concentrations 1 mg/mL and 100 ng/mL, respectively, in methanol/water (50/50, v/v). Three mixtures of isomers with a total concentration of 100 ng/mL (methanol/water, 50/50, v/v) were used: (a) 3-MMC and buphedrone (*m*/*z* 178.13); (b) 3-FMC and 4-FMC (*m*/*z* 182.10); and (c) BDB and methedrone (*m*/*z* 194.13). Isomers were mixed in the ratios: 5:95, 10:90, 25:75, 40:60, 50:50, 60:40, 75:25, 90:10, 95:5. The formation of sodium and lithium adducts was supported by the use of 0.1 mmol/L lithium chloride or sodium nitrate in analyzed mixtures.

### Cyclic Ion Mobility-Mass Spectrometry

The experiments were performed on a SELECT SERIES Cyclic IMS Q-TOF^33^ (Waters Corp., Wilmslow, U.K.) equipped with electrospray (ESI) and desorption electrospray (2D-DESI, Prosolia, with a DESI XS sprayer; Waters Corp.) in a positive mode. 0.5 mM sodium formate in propan-2-ol/water (90/10, v/v) was used for the mass calibration (*m*/*z* 50–1200). The instrumental parameters were set as follows: (1) default setting—capillary voltage 2 kV, cone voltage 10 V, source temperature 100 °C, desolvation temperature 250 °C, cone gas flow 30 L/h, desolvation gas flow 600 L/h, trap CE 6 V, transfer CE 4 V, stepwave body gradient 20 V, ion guide TW pulse height 0.4 V, trap TW pulse height 4 V, trap entrance 2 V, trap bias 2 V, trap DC −4 V, post trap gradient 3 V, post trap bias 35 V, stepwave RF 200 V, ion guide RF 300 V, driftcell RF 300 V, transfer RF 200 V, helium flow rate 120 mL/min, nitrogen flow rate 40 mL/min., nitrogen pressure in mobility cell 1.73 mbar; (2) setting for labile compounds (used for BDB and methedrone)—capillary voltage 1 kV, cone voltage 10 V, source temperature 100 °C, desolvation temperature 250 °C, cone gas flow 30 L/h, desolvation gas flow 600 L/h, trap CE 2 V, transfer CE 1 V, stepwave body gradient 10 V, ion guide TW pulse height 0.2 V, trap TW pulse height 1 V, trap entrance 1 V, trap bias 1.5 V, trap DC −4 V, post trap gradient 1.5 V, post trap bias 16 V, stepwave RF 100 V, ion guide RF 300 V, driftcell RF 200 V, transfer RF 200 V, transfer RF gain 5 V, TW static height 13 V, and wave height 10 V (eject and acquire). Cyclic TWIMS parameters for ion mobility separation are summarized in the Supporting Information, SI (Tables S1 and S2). The precursor ions were isolated in a quadrupole and fragmented in the trap cell with CE 28 V for BDB and methedrone (*m*/*z* 194.13).

Three sample introduction options were used. Analyzed solutions were directly infused into a normal flow ESI source at a flow rate of 5 μL/min. Flow injection analysis (FIA) was performed on a Waters ACQUITY UPLC I-Class system coupled to an ESI source of SELECT SERIES Cyclic IMS Q-TOF (Waters Corp., Wilmslow, U.K.) via PEEK tubing (1/16″ × 0.13 mm). Sample solutions were injected into carrying liquid (methanol/water, 50/50, v/v, 0.1 mL/min). Injection volumes were 5 and 10 μL for 3-MMC, buphedrone, 3-FMC, 4-FMC, and BDB, methedrone, respectively. For desorption electrospray, samples were deposited onto the Omni Slide Hydrophobic Arrays at 3.5 ng/mm^2^ (Prosolia, Waters Corp., Wilmslow, U.K.). Methanol/water (80/20, v/v) was used as the spray liquid at a flow rate of 2.0 μL/min. The capillary voltage was maintained at 0.75 kV, nebulizing gas pressure 11 psi, source temperature 150 °C, cone voltage 10 V. The DESI geometry was as follows: spray impact angle ∼75°, spray nozzle–inlet tube orifice ∼4 mm, inlet tube orifice–sample surface ∼0.5 mm, spray nozzle–sample surface ∼2 mm.

Data were acquired, processed, and evaluated using Masslynx v.4.2 (Software Change Note 1016, Waters Corp., Wilmslow, U.K.), a modified version of Driftscope v.2.9 (Waters Corp.), and statistics software OriginPro 2020 (OriginLab, Northampton, U.S.A.). Raw arrival times included injection time (ions were transferred into the cyclic mobility cell), drift time (ions were in the cell), and dead time (ions traveled from the cell to the TOF). ATD for specific *m*/*z* were extracted and smoothed by mean function averaging of 2 points in 1 cycle using MassLynx and exported to the OriginPro. The Composite Spectrum Regression (an application in OriginPro) was used to perform multiple linear regression of ATD profiles. The isomeric ratio was *a*_1_:*a*_2_, where *y* = *a*_0_ + *a*_1_*x*_1_ + *a*_2_*x*_2_. The intensities over the whole range of the ATD profiles of the pure isomers (1 and 2) were used as the input data *x*_1_ and *x*_2_ (independent variables). These profiles were generated as the average of six data acquisitions. The intensities over the entire ATD profile of the isomeric mixture (all six data acquisitions individually) were used as *y* (dependent variable).

Six data acquisitions were performed for each isomer and each mixture to obtain the relationships between the given and determined isomeric ratios. Model samples containing four analytes and corresponding calibration curve points were analyzed in six replicates by FIA. Outliers in the data, if found, were excluded. Ion structures, charge densities, and relative energies (Figures S1, S2, Tables S4, S5, and S6–S13, SI) were obtained for several tautomers and their conformers by a combination of Born–Oppenheimer molecular dynamics and density functional theory calculations. The structures and charge densities were used to calculate theoretical CCS, as described in the Supporting Information. Experimental values of CCS (Table S14, SI) were determined using a calibration mixture of N-ethylaniline, acetaminophen, caffeine, sulfaguanidine, and alprenolol according to the procedures described by Bush et al.,^34,35^ and McCullagh et al.^36^ Details are provided in the SI (CCS calibration, Table S3).

## Results and Discussion

### Electrospray Mass Spectra

**Figure 2:**
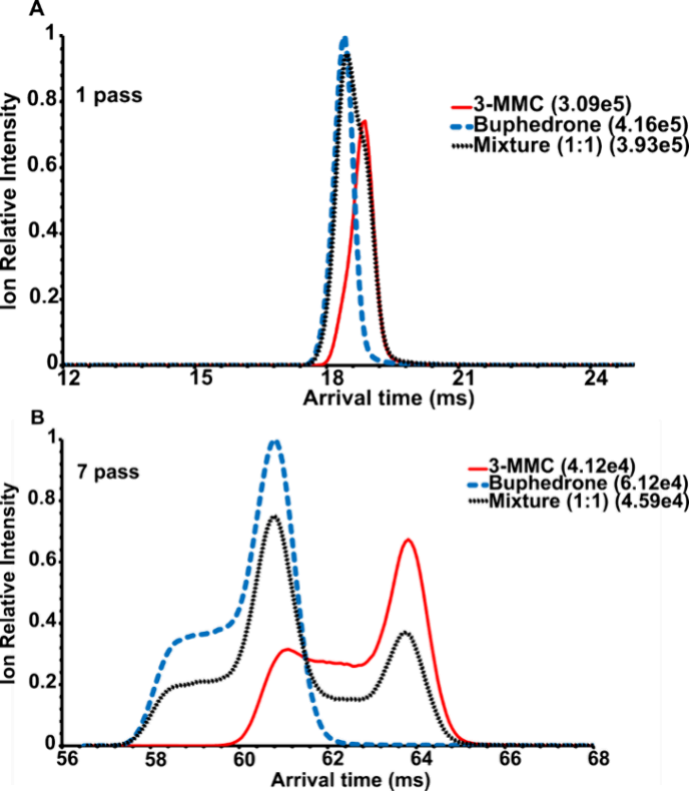
Extracted ATD profiles ([M + H]^+^, *m*/*z* 178.13) of 3-MMC, buphedrone and their mixture (50:50): (A) 1 pass, (B) 7 pass experiment. The highest absolute intensity for each mobilogram is given in brackets.

The new psychoactive substances investigated in this study (Figure 1) were easily protonated by electrospray, but isomeric ions [M + H]^+^ were not well separated by ion mobility, even in multipass experiments at higher resolving power. Nevertheless, the arrival time distribution (ATD) profiles were characteristic of the individual isomers (see, e.g., Figure 2). This is in good agreement with our previous observations using the linear and cyclic mobility cell for isomeric oligosaccharides^32^ and oligonucleotides,^37^ respectively. Due to the low intensity of the sodium adducts (Figure S3, SI), Na^+^ and Li^+^ salts were alternatively added to the analyzed solutions to support adduct formation and to allow their ion mobility analysis.

### Isomer Analysis Using Characteristic Arrival Time Distribution vs Ion Mobility Separation of Lithium Adducts

The first two isomeric pairs were analyzed using direct infusion of solutions into the electrospray. For 3-MMC and buphedrone ([M + H]^+^, *m*/*z* 178.13), single pass experiment (the path length *L* = 98 cm) was insufficient to separate their mixture (Figure 2A). This was consistent with the expected CCS for the keto and enol isomers of protonated 3-MMC (ions **1a**^**+**^ and **1b**^**+**^) and buphedrone (ions **2a**^**+**^ and **2b**^**+**^) that were in the narrow range of 139–141 Å^2^ (Figure S1, SI). The narrow range was also observed for experimental CCS values (Table S14, SI), when the percent difference in collision cross sections determined for isomers by the single pass experiment was 2%. It was estimated that the CCS-based resolving power *R*_CCS_ of about 104 was required to achieve the two-peak resolution *R*_p-p_ = 1.23 (ca. 90% separation, defined for two Gaussian peaks of equal abundance).^38^ For buphedrone, the single pass experiment gave *R*_1,CCS_ ≈ 45 (both keto- and enol-form contributed to peak width). The resolving power increases with the path length (*L*) and thus with the number of passes (*n*) *R*_*n*,CCS_ = *R*_1,CCS_ × *n*^1/2^.^33^ Six passes through the cyclic mobility cell should yield *R*_6,CCS_ ≈ 110. After the multipass experiment with seven passes (*L* = 686 cm), we were able to separate the major isomeric forms of 3-MMC and buphedrone (experimental R_7,CCS_ ≈ 126). The signals of both isomers were clearly detected in the mobilograms of their mixtures (Figure 2B). While it can be sufficient for the detection of individual isomers, simple integration of mobility peaks was not possible neither at higher resolving power, as the isomers’ ATD profiles became broader and the less abundant enol isomer of 3-MMC still significantly overlapped with buphedrone (Figure 2B). Because ATD profiles are related to extracted ion mobilograms for *m*/*z* 178.13, they reflect the migration of protonated molecules and do not include the signal of fragments such as the [MH–H_2_O]^+^ ions. Similarly ion mobility peaks of protonated molecules of isomers interfered, e.g., for cathinones and peptides separated by trapped ion mobility^30^ and cyclic TWIMS,^39^ respectively. Broadening of ion mobility signals and more complex ATD profiles of individual isomers at higher resolving power can compromise isomer separation. It can represent a more general issue in ion mobility separation of isomers existing in different conformers or protonation isomers. Such broadening can be suppressed by separating adducts of isomeric molecules with, for example, alkali metal cations. Separation of adducts has been described, e.g., for fentanyl,^29^ glycan,^40^ and flavonoid^41^ isomers. On the contrary, the broadening can be advantageous if it provides ATD profiles characteristic of individual isomers.

Since the ATD profiles of protonated 3-MMC and buphedrone differed significantly in the 7 pass experiment (Figure 2B), we performed multiple linear regression (MLR) to determine the isomeric ratio. Compared to our previous fitting procedure using Gaussian functions,^32^ the new data processing was easier to implement because MLR is the common part of routinely used software, such as OriginPro.

**Figure 3:**
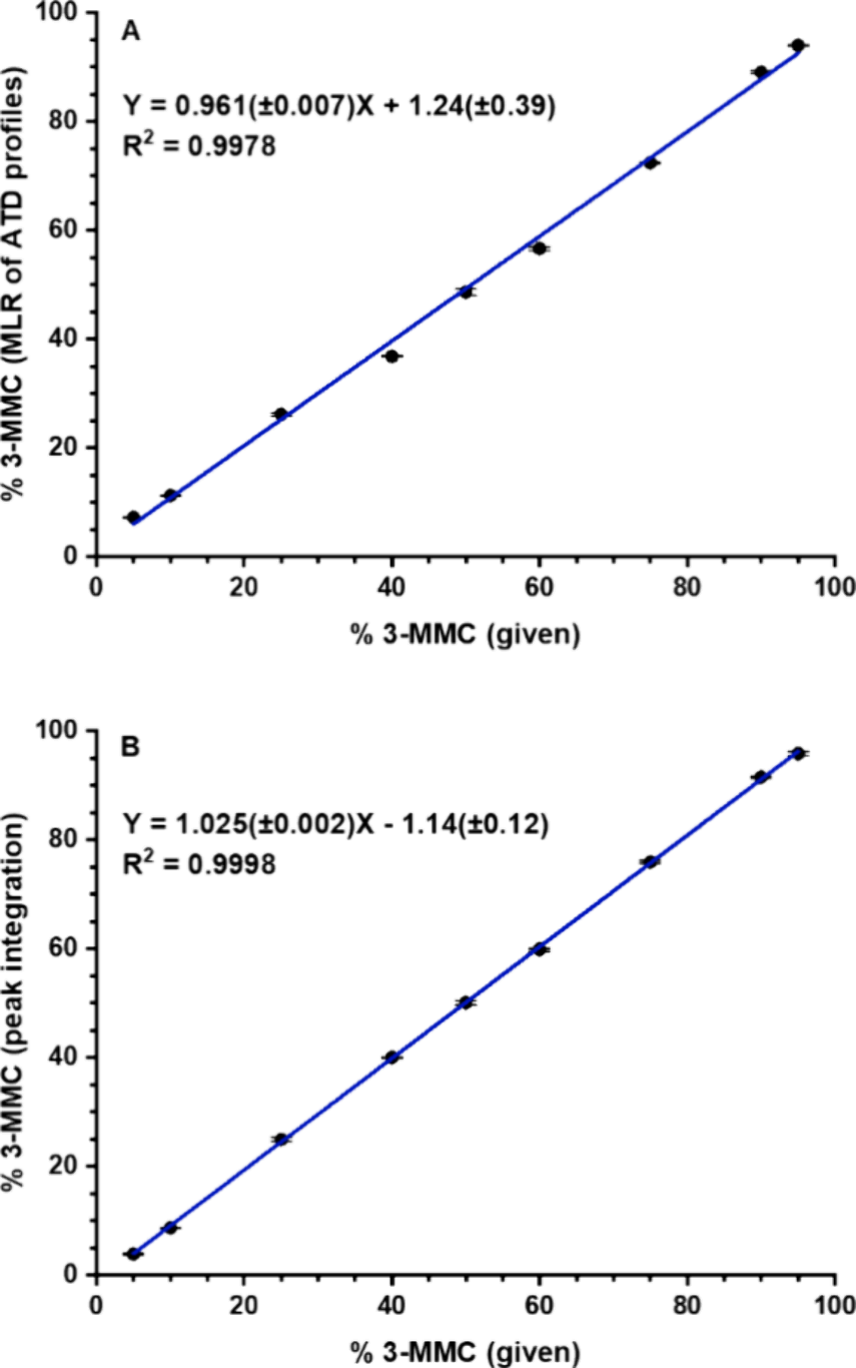
Determination of 3-MMC in the mixture with buphedrone: (A) Multiple linear regression (MLR) applied to ATD profiles of protonated molecules, seven pass separation; (B) ion mobility of lithiated molecules, ten pass separation. Standard deviations of slope and intercept are shown in brackets. Each point was measured in six data acquisitions.

The relationship between the determined and given content of 3-MMC showed good linearity (Figure 3A), proving the applicability of the proposed procedure using characteristic ATD profiles and MLR in the determination of the isomeric ratio. It is worth noting that even single pass experiment provided the linear relationship but in a limited range (5% to 75% of 3-MMC, Figure S4, SI), since buphedrone was not sufficiently detected at the excess of 3-MMC (3-MMC: buphedrone 95:5 and 90:10). It is worth noting that we observed good intraday repeatability of the ATD profiles of the isomers. When necessary, the shift in drift time was compensated by the peak alignment. Interday measurements occasionally showed a shift in the drift time and/or in the relative intensities, resulting in a change in the shape of the ATD profiles (Figure S5, SI). It might be due to the variation of experimental parameters such as drift or collision gas pressure for the same instrumental setting. If this is the case, then we recommend to acquire a complete data set during 1 day after the instrument has stabilized for 2 h after switching from standby to run mode.

**Figure 4:**
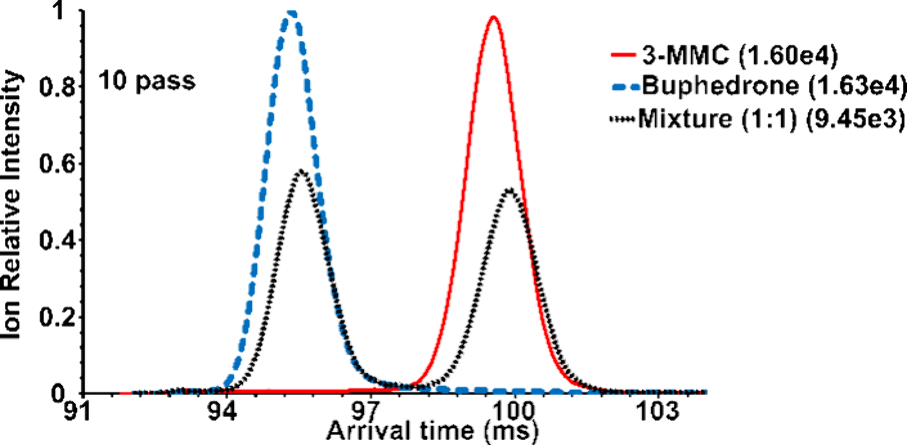
Extracted ATD profiles ([M + Li]^+^, *m*/*z* 184.13) of 3-MMC, buphedrone and their mixture (50:50), ten pass experiment. The highest absolute intensity for each mobilogram is given in brackets.

For comparison, we separated sodiated and lithiated molecules in ten passes (compare Figures 4 and S6, SI). Note that the signal intensities of the [M + Li]^+^ ions were more than one order higher than those of [M + Na]^+^. Therefore, [M + Li]^+^ ions were selected for isomeric ratio determination. Sufficient separation (Figure 4) allowed direct integration of the mobility peaks. The measured and given 3-MMC contents showed excellent linearity (Figure 3B). Cationization by these alkali metals affected the formation of ion isomers in the gas phase in that no complex peaks were observed (cf. Figures 2 and 4). This observation was consistent with the calculations of structures and CCS (Figures S1, S2, Tables S4, and S5, SI). The differences in CCS for keto (ions **1a**^**+**^ and **2a**^**+**^) and enol (ions **1b**^**+**^ and **2b**^**+**^) isomers of protonated molecules of 3-MMC and buphedrone were 0.4% and 0.6%, respectively. For both **1**^**+**^ and **2**^**+**^, the enol isomers had higher Gibbs energies than the keto forms (Table S4, SI) and thus could contribute as only minor components to the signal at *m*/*z* 178.13 in mobilograms, as manifested by peaks broadening (Figure 2B). For lithiated isomers (Figure S2, SI), the keto forms (ions **3a**^**+**^ and **4a**^**+**^) differed by 1.5% in CCS (experimental CCS (Table S14, SI) differed by 1.8% and 1.9% for 1 and 10 passes, respectively). Such a small difference highlighted the significance of multipass separation to achieve sufficient resolving power (in analogy to the protonated molecules discussed above). Enol isomers of the lithium ion adducts (ions **3b**^**+**^ and **4b**^**+**^) showed much higher energies than the keto-tautomers (Table S5, SI), and were unlikely to significantly contribute to the signal at *m*/*z* 184.13 in mobilograms. The CCS difference and isomer consistency were reflected by the well separated and symmetrical peaks of Li^+^ adducts in the ten pass mobilogram (Figure 4). The trends in Figure 3A and B are comparable, although Li^+^ adducts’ separation gave lower standard deviations of intercept and slope and slightly higher coefficient of determination than multiple linear regression using ATD profiles of protonated molecules. Both approaches represent a useful and comparable alternative to analyzing isomeric mixtures.

**Figure 5:**
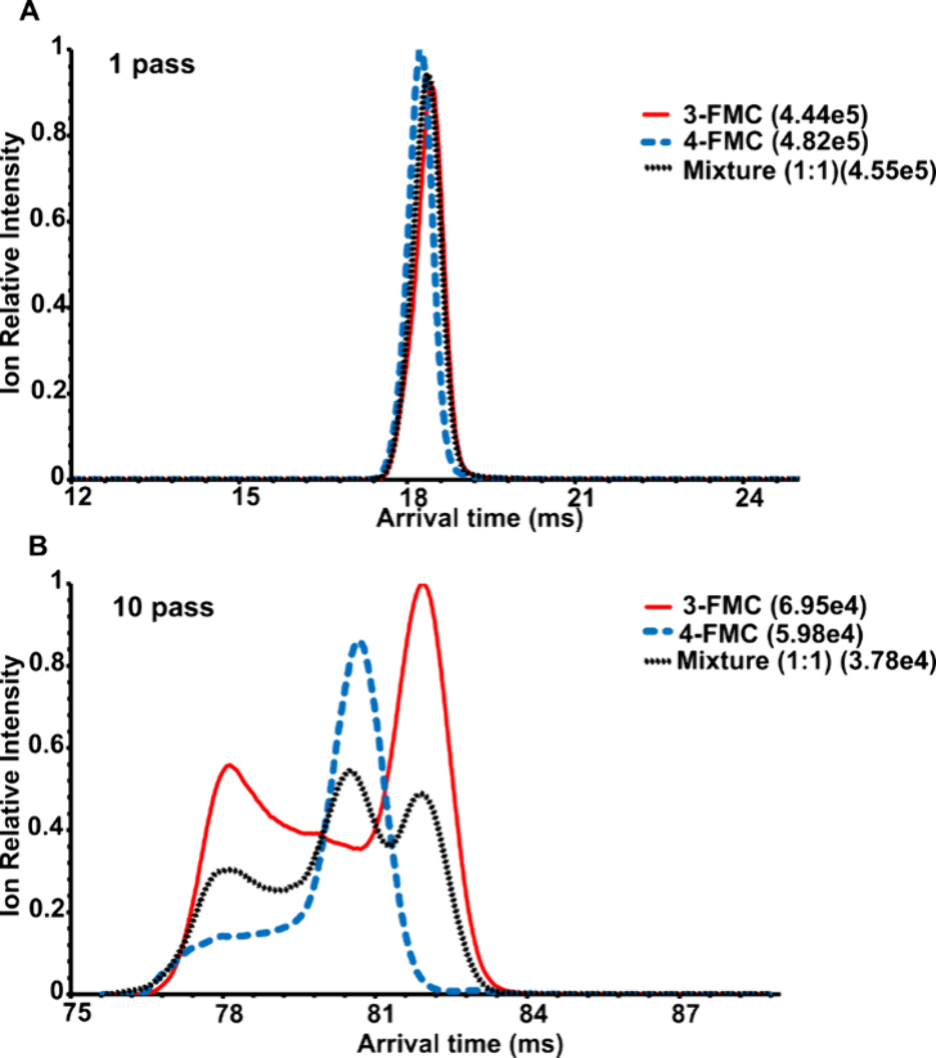
Extracted ATD profiles ([M + H]^+^, *m*/*z* 182.10) of 3-FMC, 4-FMC, and their mixture (50:50): (A) 1 pass, (B) 10 pass experiment. The highest absolute intensity for each mobilogram is given in brackets.

**Figure 6:**
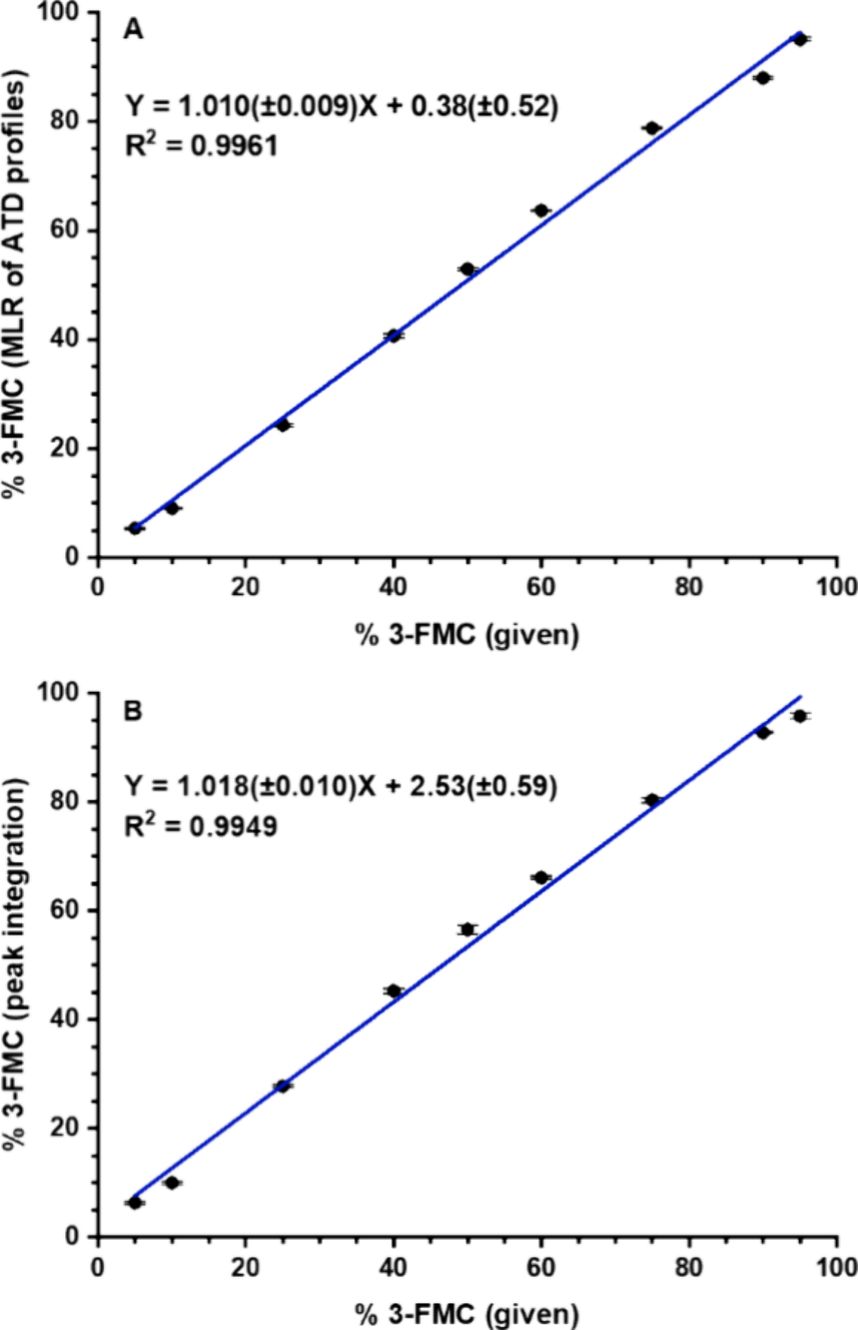
Determination of 3-FMC in the mixture with 4-FMC: (A) Multiple linear regression (MLR) applied to ATD profiles of protonated molecules, ten pass separation; (B) ion mobility of lithiated molecules, 25 pass separation. Standard deviations of slope and intercept are shown in brackets. Each point was measured in six data acquisitions.

3-FMC and 4-FMC showed symmetrical and highly overlapping ATD profiles of protonated molecules in single pass separation (Figure 5A), which did not allow us to determine the isomeric ratio even after applying multiple linear regression. In this case, higher resolving power was required, highlighting the advantage of cyclic over linear TWIMS. Ten pass separation provided characteristic ATD profiles of protonated molecules of individual isomers (Figure 5B) and the linear relationship between the determined and given content of 3-FMC in a mixture (Figure 6A). Sodium did not effectively form adducts with FMC isomers (Figure S7A, SI). Lithiated molecules were generated with sufficient intensity and partially resolved in 25 passes (Figure S7B, SI), allowing peak integration. Both approaches, MLR of ATD profiles of protonated molecules and the integration of peaks of Li^+^ adducts, were applicable. The relationship between the determined and given content of 3-FMC showed comparable standard deviations of slope and intercept and coefficients of determination (Figure 6A and 6B). Separation of adducts may be more straightforward, but MLR of ATD profiles gave similar results and may be more advantageous when adducts formation or separation is insufficient, as in the case of sodium adducts here.

### Flow Injection Analysis of Isomers Using Characteristic ATD Profiles of Fragment Ions

**Figure 7:**
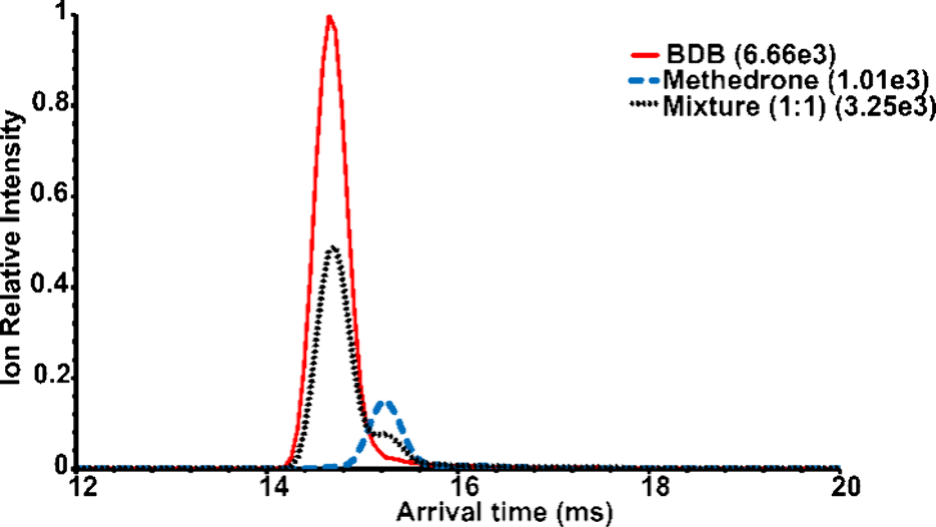
Extracted ATD profiles of fragment ions (*m*/*z* 135.04–135.08) of BDB, methedrone, and their mixture (50:50) in single pass separation. The highest absolute intensity for each mobilogram is given in brackets.

**Figure 8:**
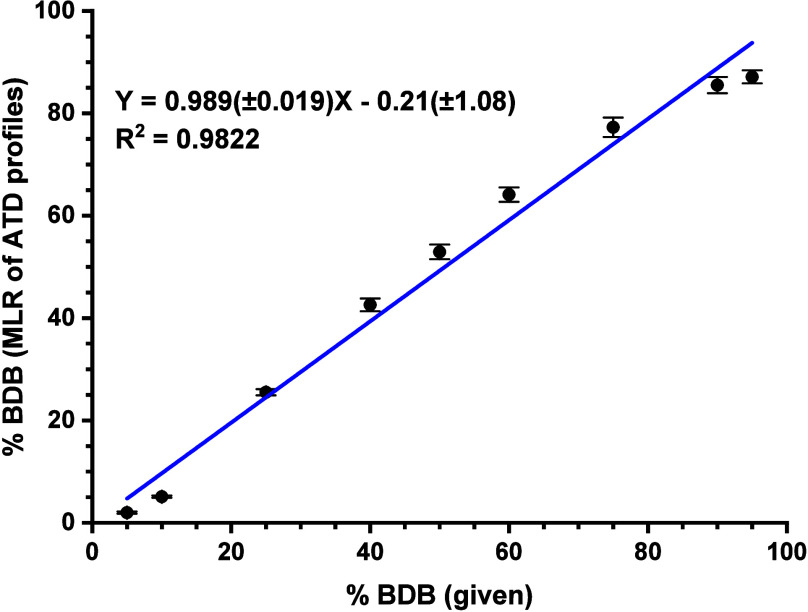
Determination of BDB in the mixture with methedrone. Multiple linear regression (MLR) applied to ATD profiles of fragment ions (*m*/*z* 135.04–135.08), single pass separation. Standard deviations of slope and intercept are shown in brackets. Each point was measured in six data acquisitions.

The third pair of isomers showed significant differences in the intensities of the [M + H]^+^ ions. In the case of BDB, this ion was found to fragment easily. While methedrone is a synthetic cathinone, BDB belongs to the phenylethylamines. The ATD profiles of both BDB and methedrone were symmetrical and completely overlapped (Figure S8, SI). The signal intensities at the maxima of the mobility peaks differed by more than 2 orders of magnitude (Figure S8A,B, SI), favoring methedrone when we used the same ion source setting (default) as for the previous isomeric pairs. Tuning the ion source and ion optics for BDB increased its signal intensity but decreased the signal intensity of methedrone. The use of three passes instead of one did not sufficiently improve separation and caused a significant decrease in BDB signal intensities (Figure S8C,D, SI). Due to unresolved symmetrical peaks with very different intensities, the use of ATD profiles of protonated molecules for isomer analysis was not possible. We proposed an alternative approach using ATD profiles of fragment ions. Both isomers fragmented to produce ions at *m*/*z* 135.04, methedrone also at *m*/*z* 135.08 (Figure S9, SI). We activated and fragmented precursor ions [M + H]^+^ in the trap section (in front of the ion mobility cell) and included both fragment ions (*m*/*z* 135.04 and 135.08) in extracted ion mobilograms (Figure 7). ATD profiles were characteristic of individual isomers and partially separated. The experiments were performed using flow injection analysis as an alternative to direct infusion, allowing for automated injections and faster data acquisition. The coefficient of determination was lower and the standard deviations at the data points were higher (Figure 8) compared to the analyses described above (Figures 3 and 6). This may be due to the more complex experiments requiring the generation and separation of fragment ions. Differentiation of isomers and even analysis of isomeric mixtures was still possible, although accuracy may be limited when one isomer is present in excess. While 5% and 10% BDB mixtures were distinguishable, the isomeric ratios determined at 90% and 95% BDB were not significantly different.

### Desorption Electrospray Ionization Generating Characteristic ATD Profiles

**Figure 9:**
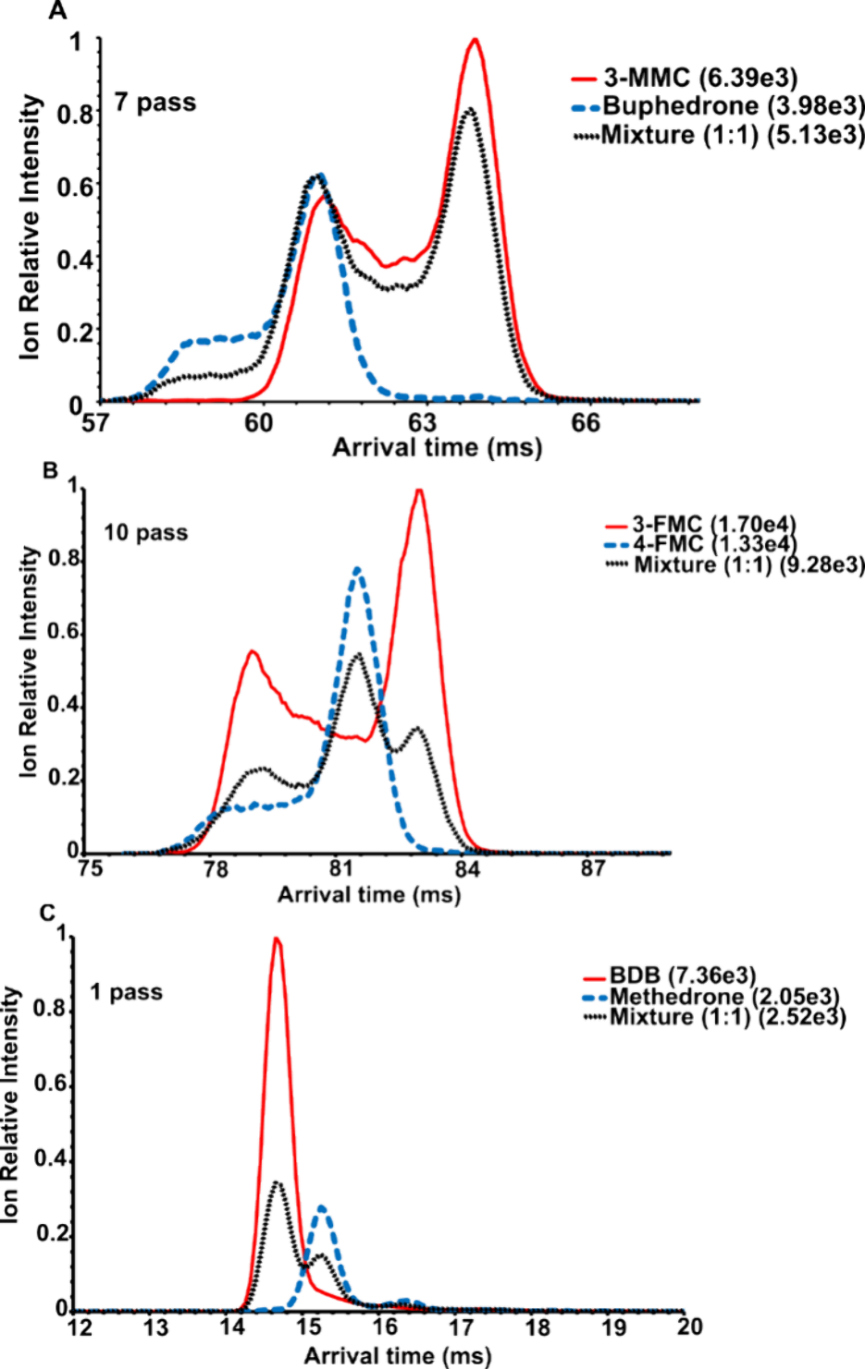
Extracted ATD profiles of individual isomers and their mixtures (50:50) desorbed and ionized by DESI: (A) 3-MMC and buphedrone ([M + H]^+^, *m*/*z* 178.13), 7 passes; (B) 3-FMC and 4-FMC ([M + H]^+^, *m*/*z* 182.10), 10 passes; (C) fragment ions (*m*/*z* 135.04–135.08) of BDB, methedrone, 1 pass. The highest absolute intensity for each mobilogram is given in brackets.

**Figure 10:**
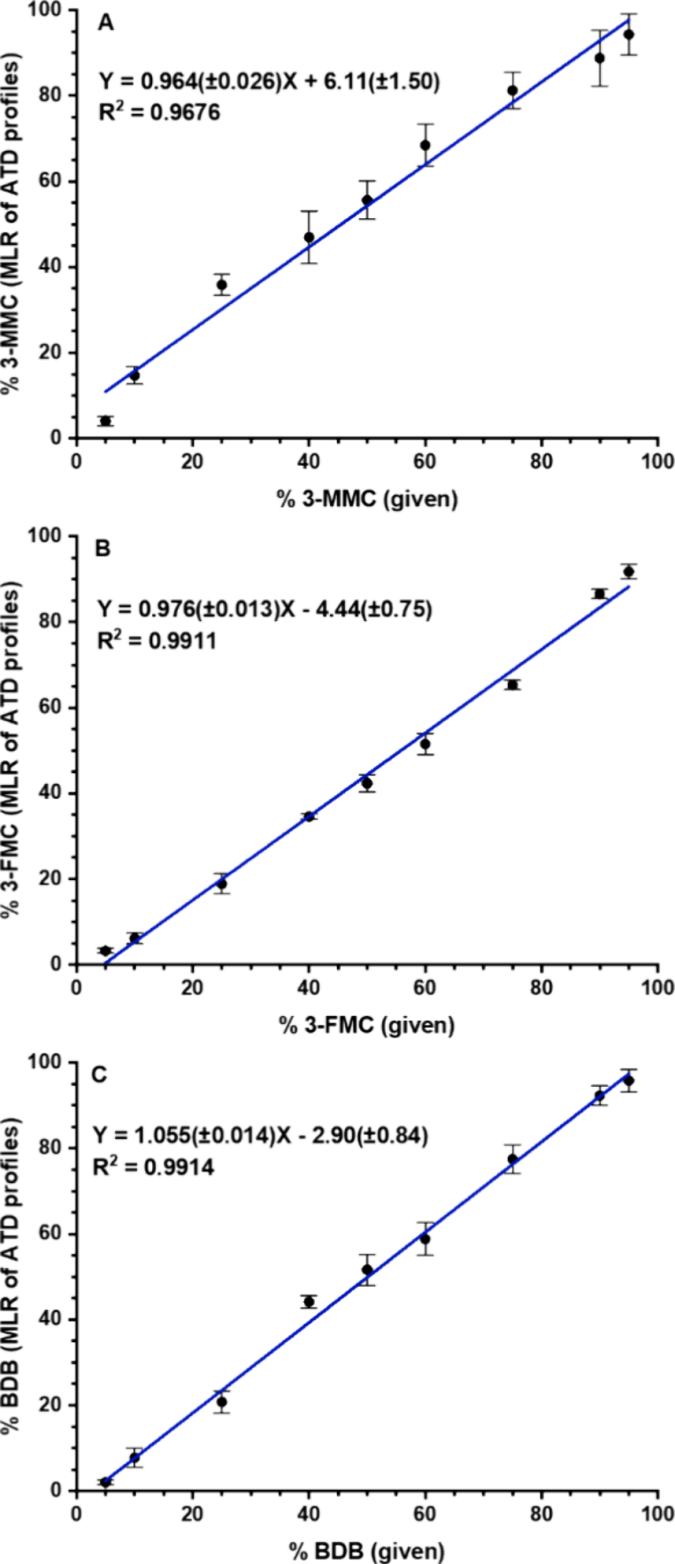
Determination of isomeric ratios using DESI and multiple linear regression (MLR) applied to ATD profiles: (A) 3-MMC in the mixture with buphedrone, ([M + H]^+^, *m*/*z* 178.13, 7 passes); (B) 3-FMC in the mixture with 4-FMC ([M + H]^+^, *m*/*z* 182.10, 10 passes); (C) BDB in the mixture with methedrone (fragment ions at *m*/*z* 135.04–135.08, 1 pass). Standard deviations of slope and intercept are shown in brackets. Each point was measured in six data acquisitions.

In analogy to the experiments described above, all three isomeric pairs ionized by DESI provided ATD profiles characteristic of isomers but with lower intensities (Figure 9). The shapes of the ATD profiles of individual isomers were similar for ESI and DESI (compare Figures 9A–C and 2B, 5B, 7). However, DESI provided different relative ionization yields for some isomers. This resulted in different shapes of ATD profiles of isomeric mixtures when comparing ESI and DESI experiments. While the shape was very similar for the 3-FMC/4-FMC mixture (Figures 9B and 5B), it was significantly different for the 3-MMC/buphedrone mixture (Figures 9A and 2B). In general, it is not possible to use ATD profiles of individual isomers obtained with one type of ion source to evaluate samples measured with another type of ion source. This is a direct consequence of the different mechanisms of ionization. DESI desorbed and ionized analytes from solid samples deposited on a target in a nonuniform layer (pipetting of solutions and drying on air), which explains the described differences and higher data variability (see error bars in Figure 10) in contrast to ESI. Evaluating data for three studied isomeric pairs, lower accuracy was found for 3-MMC/buphedrone (lower coefficient of determination, higher variability of intercept and slope, Figure 10). Nevertheless, the trend showing the increasing 3-MMC content in a mixture was clearly visible (Figure 10A). Coefficients of determination were higher than 0.99 for other two pairs (Figure 10B, C). DESI extended the applicability of our ATD profile-based approach to the analysis of solid samples.

### Rapid Determination of Isomeric Ratios in Drugs of Abuse Mixtures

**Figure 11:**
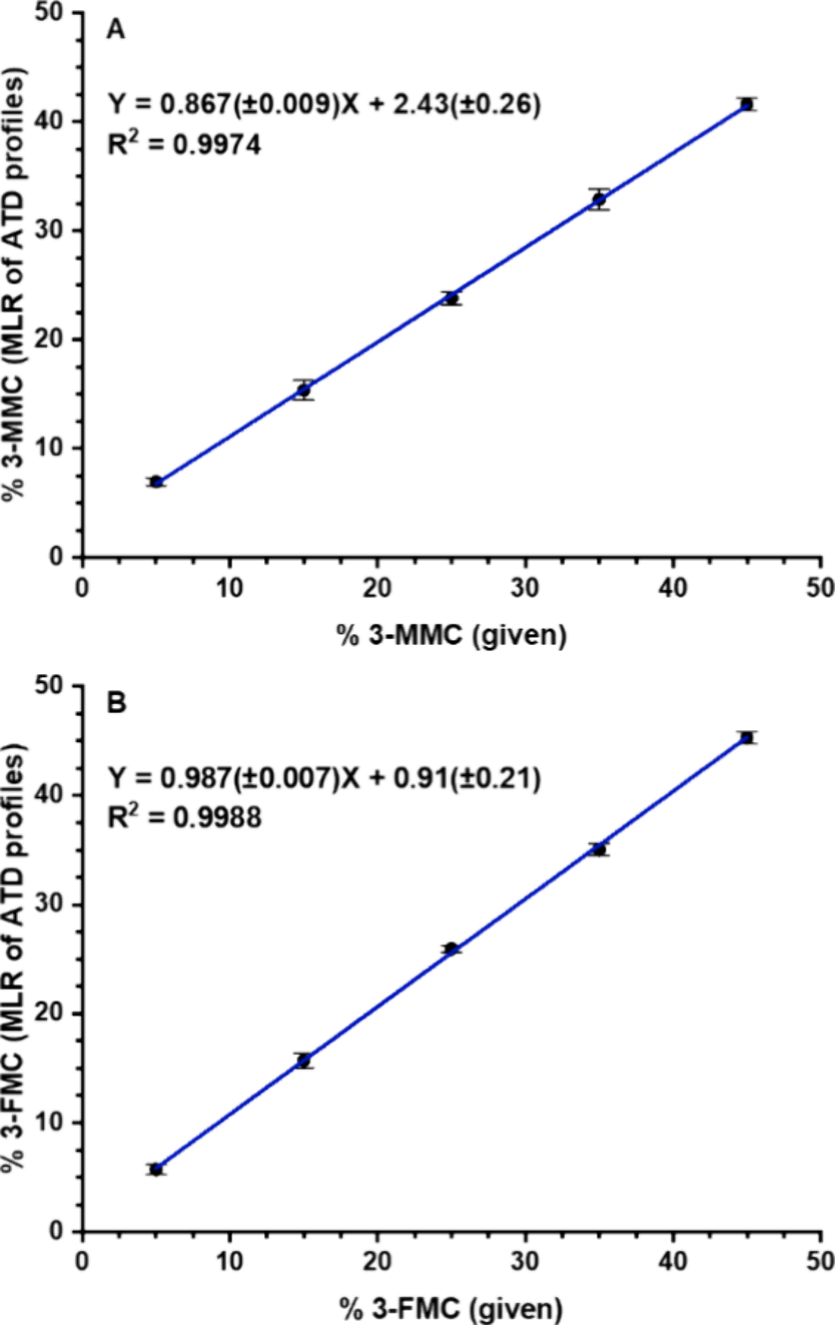
Calibration curves for FIA of two isomeric pairs mixture: (A) 3-MMC in the mixture with buphedrone, seven pass separation; (B) 3-FMC in the mixture with 4-FMC, ten pass separation. Multiple linear regression (MLR) was applied to ATD profiles of protonated molecules. Standard deviations of slope and intercept are shown in brackets. Each point was measured in six replicates.

To demonstrate the applicability of ATD profiles in isomeric mixture analysis, we performed a flow injection analysis of four drugs of abuse (two isomeric pairs) in a mixture. Data acquisition for both calibration curves and a sample of abused drugs took less than 3 h for 96 injections (six replicates of each data point) using an autosampler. Calibration curves were constructed in the minor isomer range of 5% to 45%. With coefficients of determination of 0.9974 and 0.9988, respectively, they showed good linearity and low standard deviations of slopes and intercepts (Figure 11). In a model sample containing both isomeric pairs with 10% of the minor isomer, the contents were found to be 9.3% of 3-MMC (standard deviation 0.37) and 11.0% of 3-FMC (0.42) in the mixture with buphedrone and 4-FMC, respectively. The results have demonstrated the suitability of the proposed method for the determination of isomeric ratios with sufficient accuracy and analytical run time of 1.5 min.

## Conclusions

Distinction and quantification of NPS isomers was achieved by two approaches, one relying on the separation of lithium adducts, and the other on multiple linear regression using ATD profiles characteristic of individual isomers and ATD profiles obtained for isomeric mixtures. Direct infusion experiments confirmed both approaches to be comparable. The formation of adducts by electrospray ionization required the addition of Li^+^ salt to the sample solution. The higher resolving power of cyclic TWIMS in multipass experiments improved the separation of Li^+^ adducts, whereas the mobility peaks of protonated molecules were broadened and still overlapped due to the presence of tautomers associated with individual ion structures. Similar behavior was observed, e.g., for peptides^39^ and oligonucleotides.^37^ Previously, ATD profiles were fitted by Gaussian functions and a single ATD function was generated for each isomer. These ATD functions allowed isomeric ratios of oligosaccharides to be determined by ESI-linear TWIMS.^32^ Here, the fitting was replaced by multiple linear regression which simplified the analysis.

The isomeric pair of BDB/methedrone showed significant differences in the intensities of [M + H]^+^ ions and completely overlapping mobility peaks. In this case, the ATD profiles of the fragment ions allowed us to achieve a successful analysis, proving that the proposed approach is not limited to protonated molecules. The analysis was performed by FIA as an alternative to direct infusion. Desorption electrospray was used to analyze solid samples. ATD profiles characteristic of isomers were also observed, although they differed to some extent from those obtained by electrospray. This could be attributed to the different ionization mechanisms, electrospraying of solutions vs desorption and ionization from solid samples. Finally, the rapid analysis of a mixture containing two isomeric pairs was demonstrated. FIA combined with an autosampler enabled automatic data acquisition which speeded up the analysis.

While the mean mobility values were recently used to identify cathinones while isomeric ratios have not been determined,^30^ we confirmed adequate repeatability of the ATD profiles, and included the entire ATD profiles analyzing isomeric mixtures. Although the higher resolving power of cyclic TWIMS may not allow for the complete separation of isomers, it may help to generate more distinct ATD profiles of isomers.

The ATD profile-based approach can also be used to analyze the other groups of isomeric molecules by ion mobility. While the integration of peeks requires a good separation, the described approach is applicable even for overlapping signals.

## Supporting Information Available

(PDF) The Supporting Information is available free of charge at https://pubs.acs.org/doi/10.1021/jasms.4c00127.

Instrumental settings of cyclic TWIMS, CCS calibration, M06-2X/6-31+G(d,p) optimized structures of 3-MMC and buphedrone ions with calculated CCS in nitrogen, relative Gibbs energies of protonated and lithiated molecules of 3-MMC and buphedrone, experimental CCS of 3-MMC and buphedrone, +ESI-mass spectra of analytes, determination of 3-MMC in the mixture with buphedrone (single pass separation), extracted ATD profiles on two different days, extracted ATD profiles ([M + Na]^+^) of 3-MMC, buphedrone and their mixture (50:50), extracted ATD profiles of 3-FMC, 4-FMC, and their mixture (50:50) for [M + Na]^+^ and [M+Li]^+^, extracted ATD profiles ([M + H]^+^) of BDB, methedrone, and their mixture (50:50), and +ESI mass spectra of methedrone and BDB after single pass separation (PDF)
