# Supplementary material for: Cyclic Ion Mobility of Isomeric New Psychoactive Substances Employing Characteristic Arrival Time Distribution Profiles and Adduct Separation
Source: J Am Soc Mass Spectrom. 2024 Jul 1;35(8):1733–42. doi: 10.1021/jasms.4c00127 (PMC11311522; doi:10.1021/jasms.4c00127)
Supplement: Supplementary file 1 — js4c00127_si_001.pdf [file js4c00127_si_001.pdf]

Supporting Information

Cyclic ion mobility of isomeric new psychoactive substances employing characteristic arrival time distribution profiles and adduct separation

Marianna Nytka[1], Jiahao Wan[2], František Tureček[2], Karel Lemr[1,*]

[1]Department of Analytical Chemistry, Faculty of Science, Palacký University, 17. listopadu 12, 77146, Olomouc, Czech Republic

[2]Department of Chemistry, University of Washington, Seattle, 98195-1700, United States

*Corresponding author: karel.lemr@upol.cz

Table of Contents

S.1 Instrumental settings

Table S1. Cyclic TWIMS parameters for single pass separation for all isomeric pairs and calibration

Table S2. Separation time in a multi-pass experiment

CCS calibration

Table S3. Calibration mixture

S.2 Supporting figures and tables

Figure S1. M06-2X/6-31+G(d,p) optimized structures of 3-MMC and buphedrone $[M+H]^+$ ions with calculated CCS in nitrogen.

Figure S2. M06-2X/6-31+G(d,p) optimized structures of 3-MMC and buphedrone $[M+Li]^+$ ions with calculated CCS in nitrogen.

Table S4. Relative Gibbs energies of protonated 3-MMC and buphedrone.

Table S5. Relative Gibbs energies of 3-MMC and buphedrone Li^+ adducts.

Tables S6-S13. Cartesian coordinates of the M06-2X/6-31+G(d,p) optimized geometries of ions **1a$^+$-4b$^+$**.

Table S14. Experimental CCS of 3-MMC and buphedrone.

Figure S3. +ESI-mass spectra of A) 3-MMC; B) buphedrone; C) 3-FMC; D) 4-FMC; E) BDB; F) methedrone.

Figure S4. Determination of 3-MMC in the mixture with buphedrone. Multiple linear regression (MLR) applied to ATD profiles of protonated molecules, single pass separation.

Figure S5. Extracted ATD profiles ($[M+H]^+$, m/z 178.13) of the mixture of 3-MMC and buphedrone (50 : 50) on two different days, 7 pass experiment.

Figure S6. Extracted ATD profiles ($[M+Na]^+$, m/z 200.10) of 3-MMC, buphedrone and their mixture (50 : 50), 10 pass experiment.

Figure S7. Extracted ATD profiles of 3-FMC, 4-FMC, and their mixture (50 : 50): A) $[M+Na]^+$, m/z 204.08; 10 passes; B) $[M+Li]^+$, m/z 188.11; 25 passes.

Figure S8. Extracted ATD profiles ($[M+H]^+$, m/z 194.13) of BDB, methedrone, and their mixture (50 : 50).

Figure S9. +ESI mass spectra after 1pass separation: A) methedrone; B) BDB.

S.1 Instrumental settings

Table S1. Cyclic TWIMS parameters for single pass separation for all isomeric pairs and calibration

Traveling wave parameters		Sequence			
		Parameter	Inject	Separate	Eject and Acquire
Cyclic TW Velocity (m/s)	375	Time	10	2	Automatic
Array TW Velocity (m/s)	375	Pre Array Gradient	85	85	85
TW static height (V)	15	Pre Array Bias	70	70	70
TW start height (V)	15	Array Entrance	10	30	50
TW limit height (V)	35	Wave Height	2	0	15
TW ramping rate (V/ms)	2.5	Array Offset	45	70	45
		Array Mode	Forward	Sideways	Forward Eject
		Array Exit	50	30	2
		Post Array Gradient	35	35	35
		Post Array Bias	10	10	10

Table S2. Separation time in a multi-pass experiment

Precursor ion	Number of passes	Separation time (ms)
3-MMC/Buphedrone		
$[M+H]^+$ (m/z 178.13)	7	46.5 (DI, DESI)<br>47 (FIA)
$[M+Na]^+$ (*m/z* 200.11)	5	40
	10	85
$[M+Li]^+$ (*m/z* 184.13)	5	40
	10	82
3-FMC/4-FMC		
$[M+H]^+$ (*m/z* 182.10)	5	30
	10	65.65 (DI)<br>65.95 (FIA, DESI)
$[M+Na]^+$ (*m/z* 204.08)	10	80
$[M+Li]^+$ (*m/z* 188.1)	25	204.9

Experiments: DI – direct infusion; FIA – flow injection analysis; DESI – desorption electrospray

CCS calibration

The mixture of small molecules (Table S3) at 2 μg/mL each in acetonitrile/water (1/1, v/v, 0.1 % of formic acid) was infused directly (5μL/min) into the ESI source using the default setting. Data were acquired in 1 TOF push per bin and were collected for single and multi-pass experiments. DriftScope 2.9 software (Waters Corp., Wilmslow, UK) detected peak maxima. Bin numbers were converted to the millisecond scale. Arrival times did not include injection time. Calibrant arrival times (t_a) were obtained from one and two pass experiments (98 and 196 cm path length). Drift times for one cycle were calculated as $t_d = t_a$(two passes) – t_a(one pass). The dead time was $t_0 = t_a$(one pass) – t_d. For analytes, t_d values were determined as (t_a(n passes) – t_0)/n. Multi-pass CCS calibration was performed according to the procedure described by M. McCullagh et al.[s1] Excel file provided by Waters Corp. (© Johanna Hofmann, Kevin Pagel, Fritz-Haber-Institute of the Max-Planck-Society, Berlin, Germany) was used to determine CCS values. Calibration curves were constructed for protonated (ln(CCS') = 0.3501 x ln(t_d) + 5.862, R^2 = 0.9952) and lithiated molecules (ln(CCS') = 0.3617 x ln(t_d) + 5.842, R^2 = 0.9953). CCS' is the collision cross section corrected for ion charge and reduced mass of ion and drift gas.

Table S3. Calibration mixture

Compound	m/z	z	$^{DT}CCS_{N2}$ (Å^2)$^{Ref.}$	Separation time (ms)
N-ethylaniline	122.12	1	124.50[s2]	6
Acetaminophen	152.07	1	130.40[s2]	8
Caffeine	195.10	1	136.90[s3]	10
Suflaguanidine	215.06	1	148.40[s4]	10
Alprenolol	250.20	1	157.50[s2]	12

Supporting References

(s1) McCullagh, M.; Goscinny, S.; Palmer, M.; Ujma, J. Investigations into pesticide charge site isomers using conventional IM and cIM systems. *Talanta* **2021**, *234*, 122604. DOI: 10.1016/j.talanta.2021.122604.

(s2) Campuzano, I.; Bush, M. F.; Robinson, C. V.; Beaumont, C.; Richardson, K.; Kim, H.; Kim, H. I. Structural Characterization of Drug-like Compounds by Ion Mobility Mass Spectrometry: Comparison of Theoretical and Experimentally Derived Nitrogen Collision Cross Sections. *Anal. Chem.* **2012**, *84* (2), 1026-1033. DOI: 10.1021/ac202625t.

(s3) Hines, K. M.; Ross, D. H.; Davidson, K. L.; Bush, M. F.; Xu, L. Large-Scale Structural Characterization of Drug and Drug-Like Compounds by High-Throughput Ion Mobility-Mass Spectrometry. *Anal. Chem.* **2017**, *89* (17), 9023-9030. DOI: 10.1021/acs.analchem.7b01709.

(s4) Picache, J. A.; Rose, B. S.; Balinski, A.; Leaptrot, Katrina L.; Sherrod, S. D.; May, J. C.; McLean, J. A. Collision cross section compendium to annotate and predict multi-omic compound identities. *Chem. Sci.* **2019**, *10* (4), 983-993. DOI: 10.1039/C8SC04396E.

S.2 Supporting figures and tables

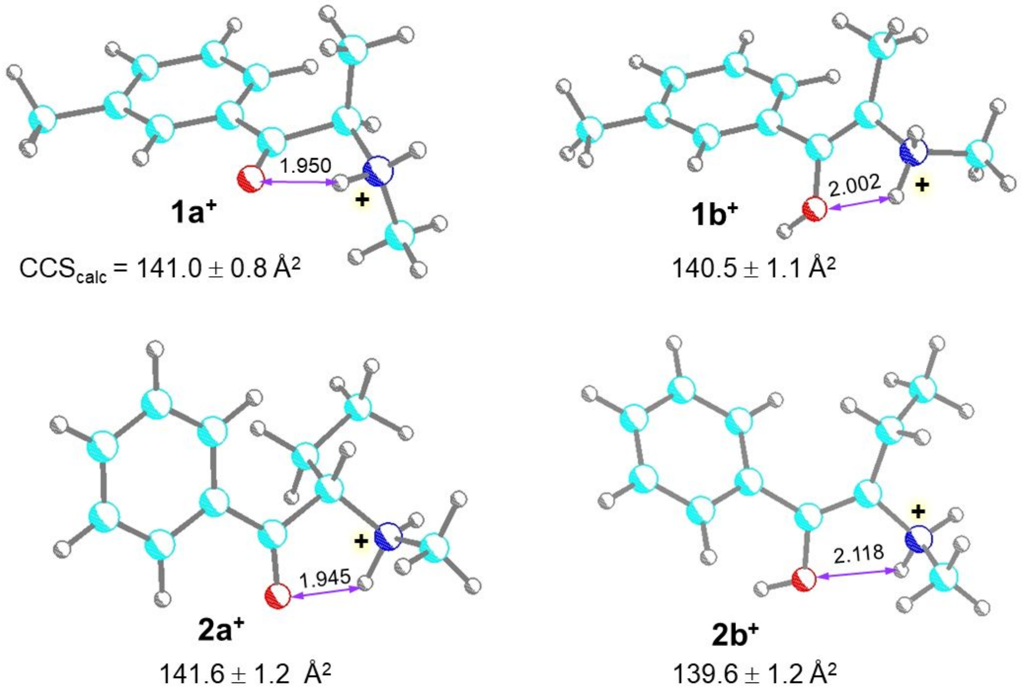

Figure S1. M06-2X/6-31+G(d,p) optimized structures of 3-MMC and buphedrone $[M+H]^+$ ions with calculated CCS in nitrogen. Atom color coding is as follows: cyan = C, blue = N, red = O, gray = H. Hydrogen bonds are shown with purple double-headed arrows with distances in Ångstrøms.

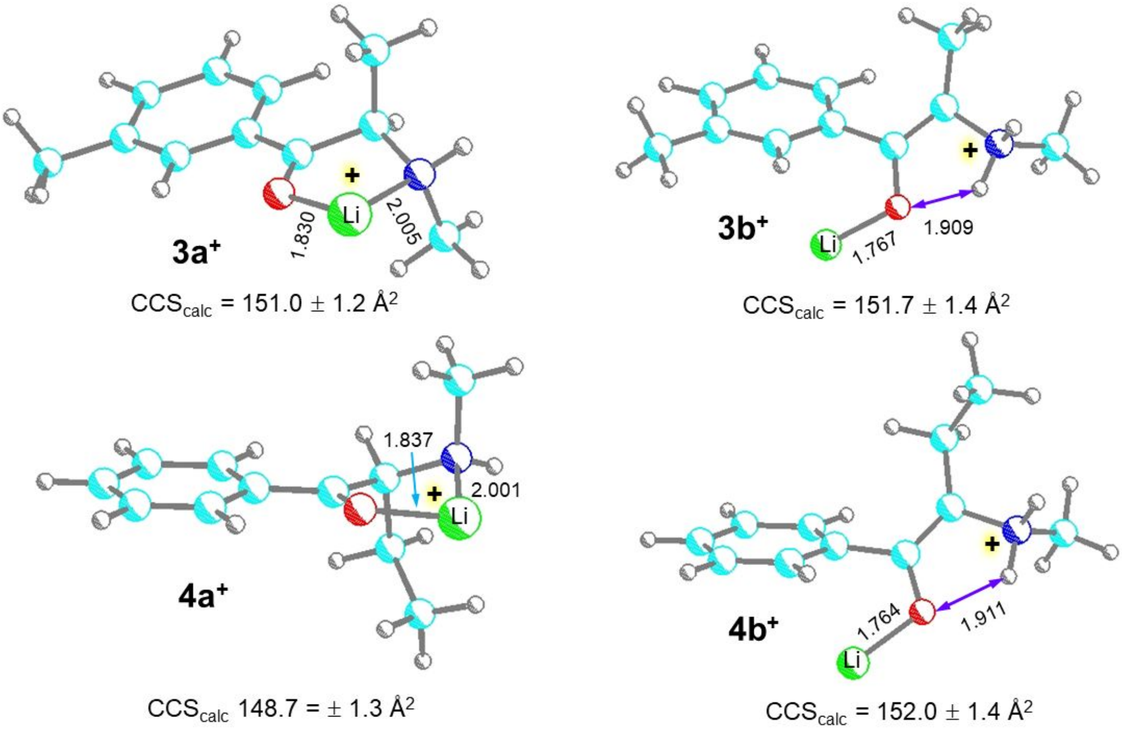

Figure S2. M06-2X/6-31+G(d,p) optimized structures of 3-MMC and buphedrone $[M+Li]^+$ ions with calculated CCS in nitrogen. C, H, N, O color coding is as in Figure S2. Hydrogen bonds are shown with ochre double-headed arrows. All bond lengths are in Ångstrøms.

Table S4. Relative energies of protonated molecules.

Ion	Relative Gibbs Energy[a,b]		
	B3LYP[c] 6-31+G(d,p)	M06-2X 6-31+G(d,p)	M06-2X[d] def2qzvpp
(3-MMC-keto + H)$^+$ (**1a**$^+$)	0.0	0.0	0.0
(buphedrone-keto + H)$^+$ (**2a**$^+$)	16	15	14
(3-MMC-enol + H)$^+$ (**1b**$^+$)	45	33	31
(buphedrone-enol + H)$^+$ (**2b**$^+$)	62	50	47

[a]In kJ mol^{-1}. [b]Including zero-point energies, enthalpies, and entropies and referring to 310 K. [c]Fully optimized with GD3-BJ empirical dispersion corrections. [d]Single-point energy calculations on M06-2X/6-31+G(d,p) optimized geometries.

Table S5. Relative energies of Li$^+$ adducts.

Ion	Relative Gibbs Energy[a]		
	B3LYP 6-31+G(d,p)	M06-2X 6-31+G(d,p)	M06-2X def2qzvpp
(3-MMC-keto + Li)$^+$ (**3a**$^+$)	0.0	0.0	0.0
(buphedrone-keto + Li)$^+$ (**4a**$^+$)	17	15	16
(3-MMC-enol + Li)$^+$ (**3b**$^+$)	82	78	75
(buphedrone-enol + Li)$^+$ (**4b**$^+$)	101	95	93

[a]Description as in Table S4.

Table S6. Cartesian coordinates of the M06-2X/6-31+G(d,p) optimized geometry of ion **1a**$^+$.

Standard orientation:

Center Number	Atomic Number	Atomic Type	Coordinates (Angstroms) X	Y	Z
1	6	0	-0.501145	0.061142	-0.015535
2	6	0	-1.562269	-0.833726	0.195504
3	6	0	-2.881550	-0.432502	0.010587
4	6	0	-3.117899	0.890256	-0.394399
5	6	0	-2.073833	1.786672	-0.609083
6	6	0	-0.758130	1.378214	-0.419782
7	6	0	-4.031150	-1.378208	0.236217
8	6	0	0.863021	-0.438400	0.197665
9	8	0	1.134348	-1.600760	0.454351
10	6	0	2.053068	0.549016	0.164201
11	6	0	2.142100	1.332861	1.471739
12	7	0	3.269771	-0.319504	0.003934
13	6	0	3.574366	-0.702462	-1.409840
14	1	0	-1.333336	-1.848168	0.508867
15	1	0	-4.142532	1.220754	-0.544454
16	1	0	-2.286827	2.801942	-0.925264
17	1	0	0.044152	2.088604	-0.596209
18	1	0	-3.679260	-2.367099	0.534718
19	1	0	-4.693889	-1.000620	1.019984
20	1	0	-4.626232	-1.487732	-0.674721
21	1	0	2.991885	2.020939	1.463936
22	1	0	1.232780	1.920061	1.611105
23	1	0	2.231371	0.651357	2.323475
24	1	0	3.037359	-1.183940	0.527560
25	1	0	4.423773	-1.384243	-1.414700
26	1	0	2.695773	-1.200577	-1.818892
27	1	0	3.805771	0.198908	-1.975978
28	1	0	2.013378	1.211489	-0.703475
29	1	0	4.083164	0.123760	0.438051

Rotational constants (GHZ): 1.6424647 0.4222286 0.3800671

Table S7. Cartesian coordinates of the M06-2X/6-31+G(d,p) optimized geometry of ion **2a**$^{+}$.

Standard orientation:

Center Number	Atomic Number	Atomic Type	Coordinates (Angstroms) X	Y	Z
1	6	0	-1.134810	-0.265645	-0.072742
2	6	0	-2.174918	-1.032772	-0.619104
3	6	0	-3.494052	-0.650621	-0.417263
4	6	0	-3.781136	0.493641	0.329968
5	6	0	-2.751043	1.259588	0.877812
6	6	0	-1.428373	0.883440	0.677537
7	6	0	0.244168	-0.716307	-0.310503
8	8	0	0.529802	-1.758671	-0.877914
9	6	0	1.427272	0.178888	0.118155
10	7	0	2.622367	-0.731204	0.114022
11	6	0	1.636311	1.329306	-0.874599
12	6	0	2.748803	2.293991	-0.463951
13	6	0	2.817437	-1.506791	1.377657
14	1	0	-1.928549	-1.917924	-1.196309
15	1	0	-4.299729	-1.240777	-0.839873
16	1	0	-4.813046	0.790236	0.487363
17	1	0	-2.980731	2.145156	1.459934
18	1	0	-0.640165	1.489972	1.114875
19	1	0	1.311476	0.554648	1.138821
20	1	0	2.428793	-1.403246	-0.651439
21	1	0	0.687843	1.868300	-0.951640
22	1	0	1.824643	0.903533	-1.868619
23	1	0	3.740811	1.827358	-0.442499
24	1	0	2.555279	2.725188	0.522458
25	1	0	2.814633	3.114896	-1.180113
26	1	0	3.011583	-0.808767	2.191120
27	1	0	1.907523	-2.075882	1.566799
28	1	0	3.659668	-2.185263	1.248326
29	1	0	3.467034	-0.199945	-0.111832

Rotational constants (GHZ): 1.3708135 0.4695843 0.4019368

Table S8. Cartesian coordinates of the M06-2X/6-31+G(d,p) optimized geometry of ion **1b**$^{+}$.

Standard orientation:

Center Number	Atomic Number	Atomic Type	Coordinates (Angstroms) X	Y	Z
1	6	0	-0.521384	0.029902	-0.158358
2	6	0	-1.589601	-0.811694	0.187009
3	6	0	-2.905005	-0.351018	0.148149
4	6	0	-3.130670	0.971939	-0.256026
5	6	0	-2.077746	1.809965	-0.609208
6	6	0	-0.765756	1.344008	-0.564051
7	6	0	-4.057918	-1.238045	0.537987
8	6	0	0.850012	-0.511190	-0.126890
9	8	0	1.050395	-1.751827	-0.660681
10	6	0	1.920636	0.098657	0.410593
11	6	0	1.992857	1.373762	1.186396
12	7	0	3.200583	-0.630275	0.255730
13	6	0	4.241798	0.107044	-0.534858
14	1	0	-1.392120	-1.827760	0.523535
15	1	0	-4.150236	1.345770	-0.296250
16	1	0	-2.278632	2.825941	-0.931985
17	1	0	0.056250	1.985173	-0.867887
18	1	0	-3.721140	-2.246112	0.787301
19	1	0	-4.581369	-0.830307	1.407436
20	1	0	-4.782690	-1.311782	-0.277468
21	1	0	2.411906	2.203306	0.607226
22	1	0	0.991402	1.662393	1.507668
23	1	0	2.603594	1.248097	2.088653
24	1	0	2.986322	-1.525047	-0.205081
25	1	0	5.126227	-0.522680	-0.627142
26	1	0	3.819249	0.328000	-1.514007
27	1	0	4.487573	1.029391	-0.011292
28	1	0	3.579557	-0.859897	1.181449
29	1	0	0.293525	-2.017921	-1.201277

Rotational constants (GHZ): 1.7765132 0.4116427 0.3636817

Table S9. Cartesian coordinates of the M06-2X/6-31+G(d,p) optimized geometry of ion **2b**$^{+}$.

Standard orientation:

Center Number	Atomic Number	Atomic Type	Coordinates (Angstroms) X	Y	Z
1	6	0	-1.056960	-0.291959	-0.130468
2	6	0	-1.556745	0.958557	-0.511618
3	6	0	-2.905145	1.250671	-0.330699
4	6	0	-3.758249	0.297079	0.224233
5	6	0	-3.265577	-0.953270	0.596609
6	6	0	-1.918456	-1.252302	0.417259
7	6	0	0.362371	-0.637244	-0.336881
8	8	0	0.642721	-1.851614	-0.897546
9	6	0	1.419789	0.119837	0.000804
10	7	0	2.730890	-0.460100	-0.374381
11	6	0	1.478968	1.415822	0.748248
12	6	0	1.985596	2.593361	-0.094999
13	6	0	3.368648	-1.286020	0.708129
14	1	0	-0.896521	1.688049	-0.971969
15	1	0	-3.292589	2.217329	-0.633927
16	1	0	-4.809376	0.527090	0.362405
17	1	0	-3.928731	-1.693072	1.031725
18	1	0	-1.531130	-2.218706	0.730030
19	1	0	2.115518	1.297054	1.635889
20	1	0	0.476639	1.626117	1.127475
21	1	0	1.992196	3.508893	0.499574
22	1	0	1.349720	2.755969	-0.968999
23	1	0	3.012912	2.447013	-0.450311
24	1	0	3.489455	-0.661158	1.591385
25	1	0	2.698323	-2.117926	0.917677
26	1	0	4.336234	-1.644727	0.357489
27	1	0	3.364661	0.295526	-0.653977
28	1	0	-0.145364	-2.219853	-1.321828
29	1	0	2.584608	-1.061186	-1.195303

Rotational constants (GHZ): 1.3827636 0.4969514 0.3979405

Table S10. Cartesian coordinates of the M06-2X/6-31+G(d,p) optimized geometry of ion **3a**$^{+}$.
Standard orientation:

Center Number	Atomic Number	Atomic Type	Coordinates (Angstroms) X	Y	Z
1	6	0	-0.545552	0.097087	-0.001594
2	6	0	-1.595939	-0.828598	0.129315
3	6	0	-2.920785	-0.437874	-0.029921
4	6	0	-3.179119	0.910031	-0.323640
5	6	0	-2.149884	1.839133	-0.455082
6	6	0	-0.827938	1.439744	-0.296215
7	6	0	-4.054681	-1.419208	0.106604
8	6	0	0.824891	-0.386985	0.185443
9	8	0	1.059234	-1.582736	0.411112
10	6	0	2.013441	0.579847	0.182561
11	6	0	2.115576	1.230178	1.567629
12	7	0	3.242616	-0.181734	-0.121084
13	6	0	3.427547	-0.370683	-1.577675
14	1	0	-1.354513	-1.861762	0.359719
15	1	0	-4.208921	1.233996	-0.451645
16	1	0	-2.379384	2.874078	-0.683742
17	1	0	-0.038672	2.175987	-0.404835
18	1	0	-3.687320	-2.421192	0.335056
19	1	0	-4.735874	-1.114918	0.906230
20	1	0	-4.634935	-1.471394	-0.818926
21	1	0	2.941515	1.946002	1.588481
22	1	0	1.196700	1.763702	1.817861
23	1	0	2.286830	0.472260	2.338900
24	3	0	2.829066	-1.999983	0.615490
25	1	0	4.370311	-0.887997	-1.764961
26	1	0	2.609329	-0.979844	-1.973834
27	1	0	3.436442	0.582040	-2.117978
28	1	0	1.859052	1.356098	-0.576757
29	1	0	4.034711	0.350872	0.234093

Rotational constants (GHZ): 1.4907024 0.4057202 0.3631808

Table S11. Cartesian coordinates of of the M06-2X/6-31+G(d,p) optimized geometry ion **4a**$^+$.

Standard orientation:

Center Number	Atomic Number	Atomic Type	Coordinates (Angstroms) X	Y	Z
1	6	0	1.078686	-0.192735	0.090673
2	6	0	1.971998	-0.614215	1.090403
3	6	0	3.339858	-0.482979	0.900051
4	6	0	3.826861	0.070656	-0.286266
5	6	0	2.947543	0.492543	-1.284444
6	6	0	1.576726	0.361640	-1.100801
7	6	0	-0.358408	-0.347513	0.341333
8	8	0	-0.772260	-0.870223	1.385299
9	6	0	-1.397592	0.204144	-0.636849
10	7	0	-2.670691	-0.512997	-0.423127
11	6	0	-1.574177	1.717097	-0.381464
12	6	0	-2.071153	2.063101	1.022618
13	6	0	-2.681050	-1.827379	-1.102348
14	1	0	1.574999	-1.040340	2.005406
15	1	0	4.028824	-0.809169	1.671359
16	1	0	4.897122	0.172627	-0.434399
17	1	0	3.331805	0.919235	-2.204260
18	1	0	0.909651	0.691144	-1.890575
19	1	0	-1.052183	0.056723	-1.667571
20	1	0	-2.272730	2.100007	-1.134125
21	1	0	-0.616663	2.213151	-0.565521
22	1	0	-1.370247	1.737227	1.798005
23	1	0	-3.061102	1.634079	1.224842
24	1	0	-2.183623	3.143980	1.126153
25	1	0	-1.918689	-2.473270	-0.657338
26	1	0	-3.656262	-2.299674	-0.972133
27	1	0	-2.472974	-1.737479	-2.174136
28	1	0	-3.417301	0.055848	-0.820149
29	3	0	-2.601157	-0.825821	1.552167

Rotational constants (GHZ): 1.3263148 0.4424122 0.4311162

Table S12. Cartesian coordinates of the M06-2X/6-31+G(d,p) optimized geometry of ion **3b**$^{+}$.

Standard orientation:

Center Number	Atomic Number	Atomic Type	Coordinates (Angstroms) X	Y	Z
1	6	0	-0.484680	0.103232	-0.106716
2	6	0	-1.548725	-0.712947	0.323908
3	6	0	-2.878780	-0.292128	0.227111
4	6	0	-3.131171	0.966818	-0.335138
5	6	0	-2.088419	1.780697	-0.765510
6	6	0	-0.763335	1.354138	-0.657453
7	6	0	-4.009918	-1.139937	0.747312
8	6	0	0.897953	-0.466129	-0.076222
9	8	0	1.033957	-1.657509	-0.624635
10	6	0	1.955964	0.187779	0.465332
11	6	0	2.050508	1.483500	1.200179
12	7	0	3.198695	-0.596887	0.322247
13	6	0	4.242949	0.021853	-0.548693
14	1	0	-1.324109	-1.656698	0.826285
15	1	0	-4.157373	1.312006	-0.425802
16	1	0	-2.306903	2.751357	-1.198829
17	1	0	0.048400	1.980993	-1.014508
18	1	0	-3.674145	-2.146377	1.007079
19	1	0	-4.438671	-0.689564	1.647523
20	1	0	-4.812211	-1.221484	0.009282
21	1	0	2.570290	1.363951	2.159379
22	1	0	2.569601	2.266196	0.634537
23	1	0	1.047105	1.849463	1.424339
24	1	0	2.860533	-1.486432	-0.095346
25	1	0	5.096222	-0.652352	-0.624116
26	1	0	3.797031	0.189887	-1.528062
27	1	0	4.550193	0.970001	-0.109318
28	1	0	3.599912	-0.811258	1.240690
29	3	0	-0.514157	-2.100886	-1.352148

Rotational constants (GHZ): 1.5531459 0.4048271 0.3597366

Table S13. Cartesian coordinates of the M06-2X/6-31+G(d,p) optimized geometry of ion **4b**$^{+}$.

Standard orientation:

Center Number	Atomic Number	Atomic Type	Coordinates (Angstroms) X	Y	Z
1	6	0	1.039779	-0.193871	-0.045456
2	6	0	1.377169	0.914032	0.737756
3	6	0	2.706984	1.320169	0.842468
4	6	0	3.713458	0.620337	0.177491
5	6	0	3.389111	-0.486775	-0.605995
6	6	0	2.059001	-0.894980	-0.713916
7	6	0	-0.352154	-0.739762	-0.098459
8	8	0	-0.475075	-2.016777	0.199546
9	6	0	-1.431412	0.013694	-0.427732
10	7	0	-2.673879	-0.789652	-0.364516
11	6	0	-1.540372	1.413439	-0.945783
12	6	0	-2.371521	2.373370	-0.084662
13	6	0	-3.357518	-0.788833	0.969865
14	1	0	0.596968	1.447545	1.273734
15	1	0	2.959380	2.180621	1.453473
16	1	0	4.746137	0.940965	0.263294
17	1	0	4.165010	-1.017618	-1.148311
18	1	0	1.794410	-1.711643	-1.387373
19	1	0	-1.952890	1.398068	-1.966019
20	1	0	-0.522357	1.798154	-1.050478
21	1	0	-2.323801	3.386476	-0.490306
22	1	0	-2.003588	2.398451	0.945611
23	1	0	-3.430360	2.092967	-0.062024
24	1	0	-3.621320	0.234297	1.228014
25	1	0	-4.247113	-1.417887	0.918751
26	1	0	-2.643601	-1.190482	1.687108
27	1	0	-2.365758	-1.758682	-0.546226
28	3	0	1.098911	-2.640263	0.701006
29	1	0	-3.325234	-0.503576	-1.100480

Rotational constants (GHZ): 1.2694629 0.4785842 0.3882588

Supporting Calculations

Ion structures were obtained by applying Born-Oppenheimer molecular dynamics (BOMD) trajectory calculations of several initial conformers, tautomers, and protonation isomers that were run for 20 ps at 510 K with Berendsen thermostat to stabilize energies. This was followed by gradient geometry optimization with density functional theory using M06-2X[s5] and the 6-31+G(d,p) basis set of selected low-energy structures, as described recently.[s6] No other protonation isomers than those displayed in Figure S2 were found as local low-energy minima in the gas phase. The lithium ion adducts showed multiple conformers for keto and enol tautomers that were local energy minima. The lowest-energy structures for each Li-ion tautomer are shown in Figure S3. The Cartesian coordinates of the M06-2X/6-31+G(d,p) optimized structures are given in Tables S6-S13. Charge distributions were calculated according to Merz, Singh, and Kollman (MK).[s7,s8] Collision cross sections (CCS) in nitrogen were calculated by the modified ion trajectory method ($MobCal_{MPI}$)[s9-s11] using the MK charge densities. The reported CCS and standard deviations are from averaging data from ten trajectory runs.

Supporting References

(s5) Zhao, Y.; Truhlar, D. G. The M06 Suite of Density Functionals for Main Group Thermochemistry, Thermochemical Kinetics, Noncovalent Interactions, Excited States, and Transition Elements: Two New Functionals and Systematic Testing of Four M06-Class Functionals and 12 Other Functionals. *Theor. Chem. Acc.* **2008**, *120*, 215-241. DOI: 10.1007/s00214-007-0310-x.

(s6) Wan, J; Nytka, M.; Vu, K.; Qian, H.; Lemr, K.; Turecek, F. Nitrile Imines as Peptide and Oligonucleotide Photocrosslinkers in Gas-Phase Ions. *J. Am. Soc. Mass Spectrom.* **2024**, *35*, 344-356. DOI: 10.1021/jasms.3c00379.

(s7) Singh, U. C.; Kollman, P. A. An Approach to Computing Electrostatic Charges for Molecules. *J. Comput. Chem.* **1984**, *5*, 129-145. DOI: 10.1002/jcc.540050204.

(s8) Besler, B. H.; Merz, K. M., Jr., Kollman, P. Atomic Charges Derived from Semiempirical Methods. *J. Comput. Chem.* **1990**, *11*, 431-439. DOI: 10.1002/jcc.540110404.

(s9) Ieritano, C.; Crouse, J.; Campbell, J. L.; Hopkins, W. S. A Parallelized Molecular Collision Cross Section Package with Optimized Accuracy and Efficiency. *Analyst* **2019**, *144*, 1660−1670. DOI: 10.1039/c8an02150c.

(s10) Ieritano, C.; Hopkins, W. S. Assessing Collision Cross Section Calculations Using MobCal-MPI with a Variety of Commonly Used Computational Methods. *Mater. Today Commun.* **2021**, *27*, 102226. DOI: 10.1016/j.mtcomm.2021.102226.

(s11) Haack, A.; Ieritano, C.; Hopkins, W. S. MobCal-MPI 2.0: an Accurate and Parallelized Package for Calculating Field-Dependent Collision Cross Sections and Ion Mobilities. *Analyst* **2023**, 148, 3257-3273. DOI: 10.1039/d3an00545c.

Table S14: Experimental CCS (Å^2) of 3-MMC and buphedrone.

Ion	single pass	multi-pass[a]
(3-MMC + H)$^+$	145.0	144.8[b]/142.0[c]
(buphedrone + H)$^+$	142.1	141.9[b]/140.2[c]
(3-MMC + Li)$^+$	154.0	153.3
(buphedrone + Li)$^+$	151.2	150.4

[a]multi-pass experiment: seven and ten passes for protonated and lithiated molecules, respectively. [b]major peak (keto-form) and [c]side peak (enol-form) in Figure 2B, their insufficient separation and possible keto-enol interconversion can affect CCS determination.

Note: For (buphedrone + H)$^+$, the experimental CCS 141.2 Å^2 has been published.[s12]

Supporting Reference

(s12) Lian, R.; Zhang, F.; Zhang, Y.; Wu, Z.; Ye, H.; Ni, C.; Lv, X.; Guo, Y. Ion mobility derived collision cross section as an additional measure to support the rapid analysis of abused drugs and toxic compounds using electrospray ion mobility time-of-flight mass spectrometry. Anal. Methods **2018**, *10* (7), 749-756. DOI: 10.1039/C7AY02808C.

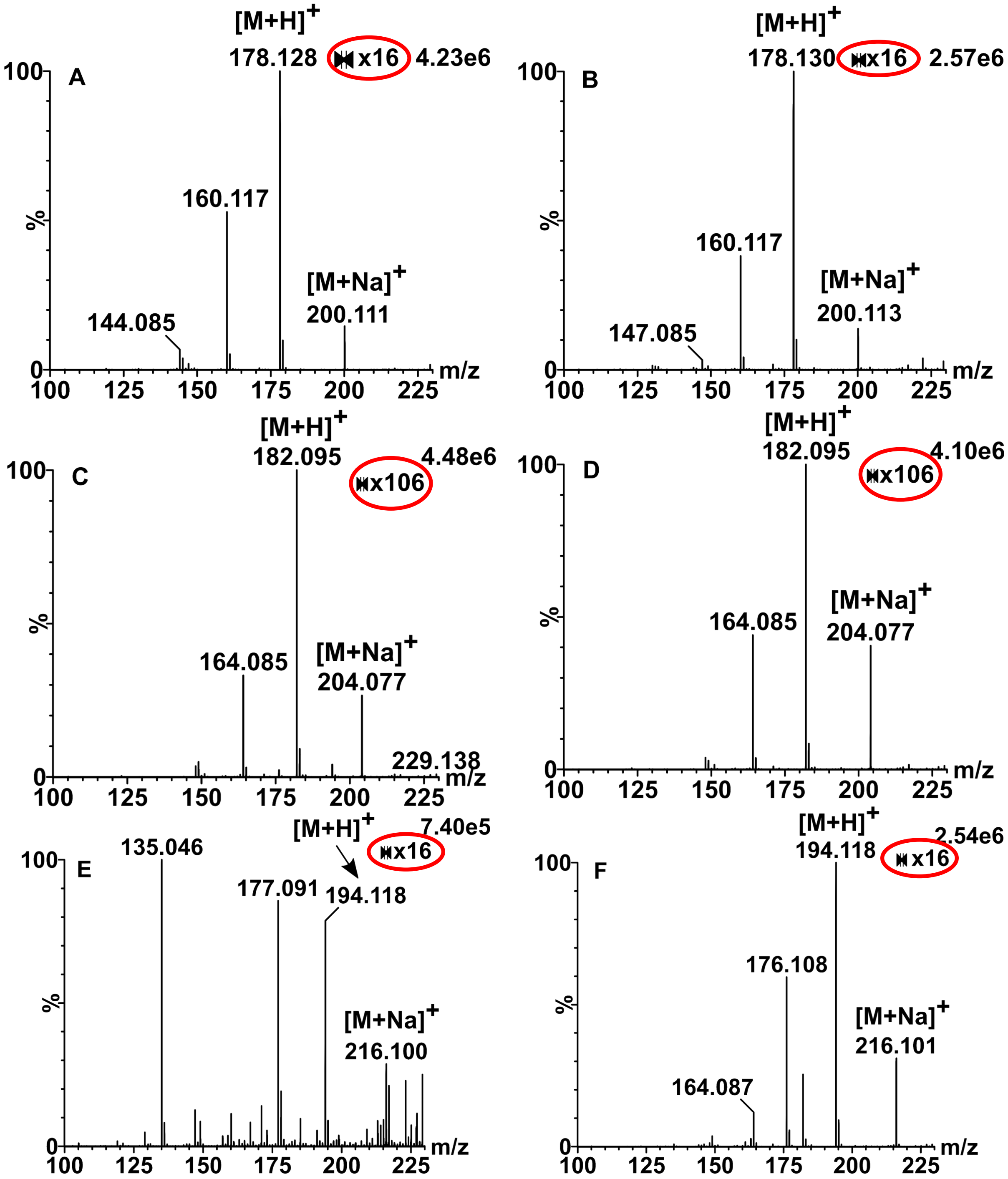

Figure S3. +ESI-mass spectra: A) 3-MMC; B) buphedrone; C) 3-FMC; D) 4-FMC; E) BDB; F) methedrone. Compared to the protonated molecules, the sodium adducts show signal intensities lower by more than one (A, B, E, F) and two (C, D) orders of magnitude (see zoom ranges marked in red).

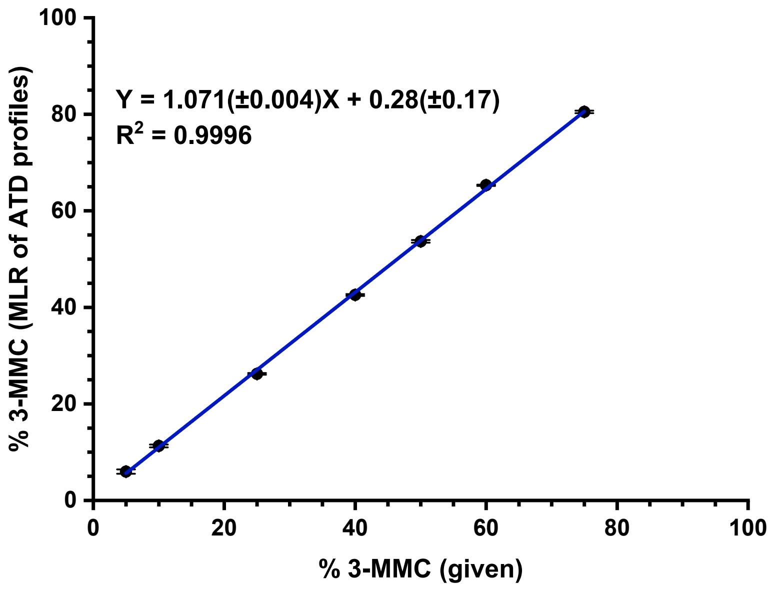

Figure S4. Determination of 3-MMC in the mixture with buphedrone. Multiple linear regression (MLR) applied to ATD profiles of protonated molecules, single pass separation. Standard deviations of slope and intercept are given in brackets. Each point was measured in six data acquisitions. The range of the relationship was limited to 5 % - 75 % of 3-MMC because the analysis of the mixtures 95 : 5 and 90 : 10 failed. The determined ratios were 100 : 0 and 99.4 : 0.6, respectively, and all values for these two mixtures were evaluated as the outliers from the linear relationship.

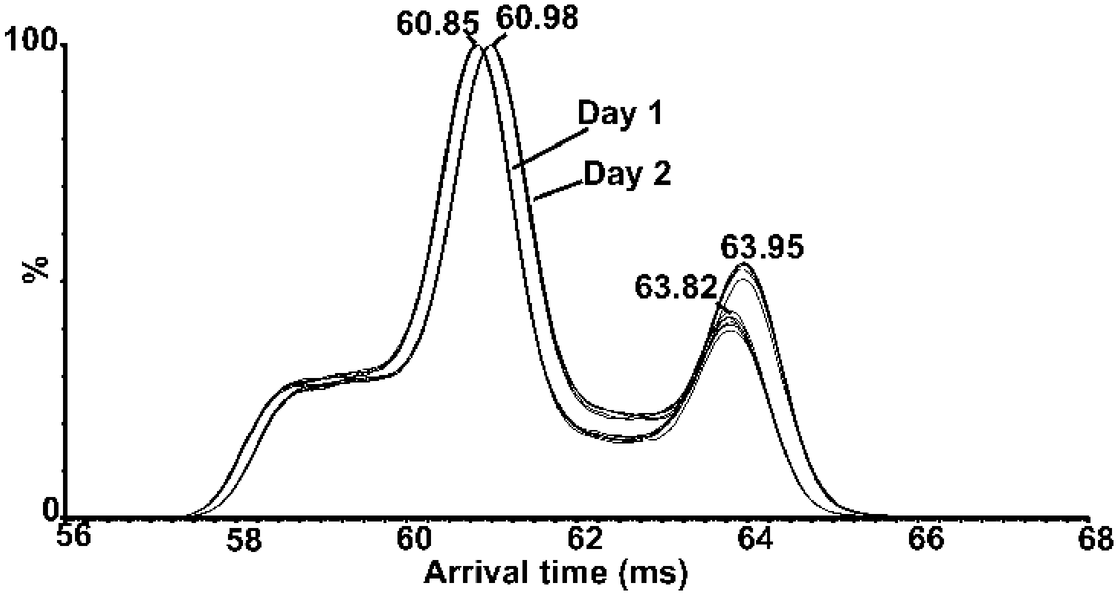

Figure S5. Extracted ATD profiles ($[M+H]^+$, m/z 178.13) of the mixture of 3-MMC and buphedrone (50 : 50) on two different days, 7 pass experiment.

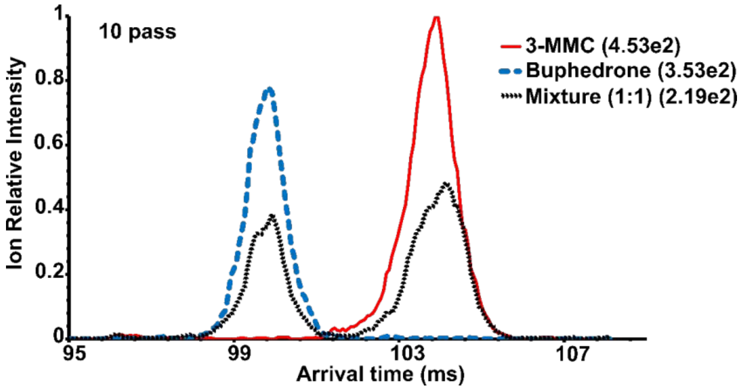

Figure S6. Extracted ATD profiles ([M+Na]$^+$, m/z 200.10) of 3-MMC, buphedrone and their mixture (50 : 50), 10 pass experiment. The highest absolute intensity for each mobilogram is given in brackets.

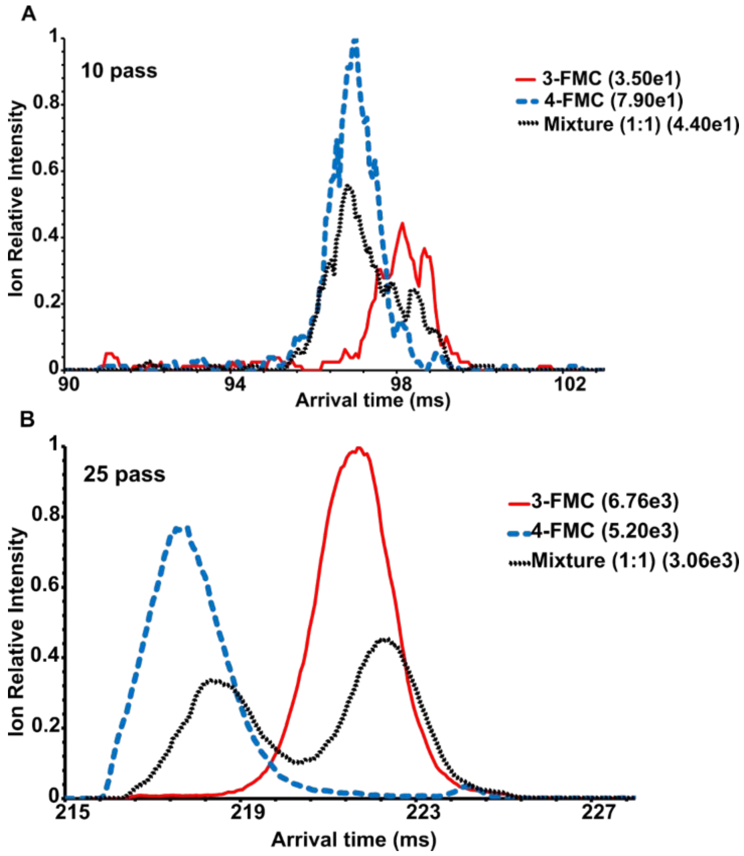

Figure S7. Extracted ATD profiles of 3-FMC, 4-FMC, and their mixture (50 : 50): A) [M+Na]$^+$, m/z 204.08; 10 passes; B) [M+Li]$^+$, m/z 188.11; 25 passes. The highest absolute intensity for each mobilogram is given in brackets.

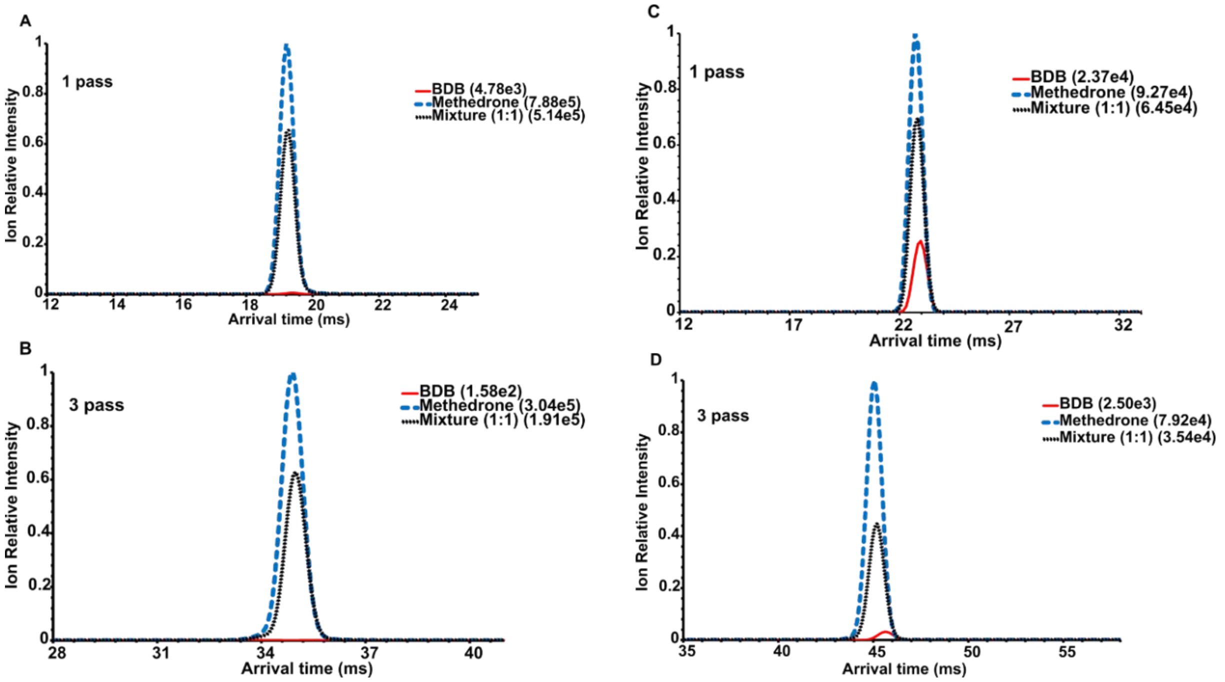

Figure S8. Extracted ATD profiles ([M+H]$^+$, m/z 194.13) of BDB, methedrone, and their mixture (50 : 50): A) default setting, 1 pass; B) default setting, 3 passes; C) setting for labile compounds, 1 pass; D) setting for labile compounds, 3 passes. The highest absolute intensity for each mobilogram is given in brackets.

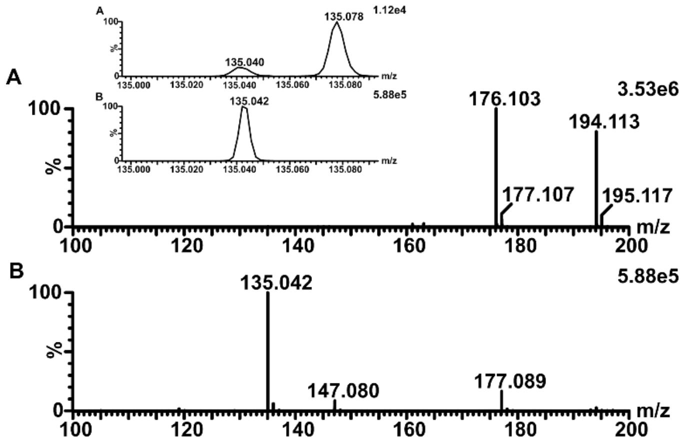

Figure S9. +ESI mass spectra after 1 pass separation: A) methedrone; B) BDB. The inset shows the zoomed detail of the signal of the fragment ions (m/z 135).
